# Health literacy among early adolescents in Umbria, Italy: a cross-sectional study

**DOI:** 10.3389/fpubh.2026.1925432

**Published:** 2026-10-27

**Authors:** Carlotta Bertola, Vito Cerabona, Leonardo Bronzetti, Francesco Paciosi, Giancarlo Pocetta, Ilaria Valentini, Manuela Chiavarini, Chiara de Waure

**Affiliations:** 1 Department of Medicine and Surgery, University of Perugia, Perugia, Italy; 2 Department of Health Sciences, University of Florence, Florence, Italy

**Keywords:** adolescents, health education, health literacy, health promotion, school health

## Abstract

**Introduction:**

Health literacy represents individuals’ ability to understand and use health information. It is related to health behaviors and outcomes; therefore, its monitoring is important. This analysis investigated health literacy among Italian adolescents using data collected within a broader study on adolescents’ vaccination and its determinants.

**Methods:**

A secondary analysis of a broader cross-sectional study on adolescent vaccination was conducted. The online survey ran from 23 April to 9 June 2024 in lower secondary schools in Umbria. The Regional School Office contacted all 72 schools; 18 participated (25.0%). The questionnaire was distributed through schools’ electronic registers to third-year students and their parents. Of 6,996 eligible adolescents, 340 completed the mandatory 29-item MOHLAA-Q (4.9%). The Italian MOHLAA-Q was translated by authors. No independent validation, back-translation, or cognitive testing was performed; the original instrument was validated in adolescents aged 14–17 years. Descriptive analyses used exploratory proportional-odds ordinal logistic regression with school-clustered standard errors.

**Results:**

Among 340 adolescents, 53.3% reported some and 17.6% many difficulties in dealing with health-related information (Scale A), while 25.3% reported few and 3.8% barely/no difficulties. For communication and interaction skills (Scale B), 8.2% were classified as low, 28.5% moderate, 31.2% rather high, and 32.1% high. For attitudes toward health and health information (Scale C), 2.9% were passive, 45.9% partly passive–partly active, and 51.2% active. For health-related knowledge (Scale D), 51.5% were classified as low, 40.9% moderate, and 7.6% high. Exploratory ordinal analyses identified associations with selected adolescent and family characteristics, including adolescent sex for Scale A and parental health literacy for Scale D; findings were considered hypothesis-generating.

**Conclusion:**

In this opportunistic, non-representative regional sample, difficulties in dealing with health-related information and low health-related knowledge were relatively common, while communication skills and attitudes showed more favorable distributions. Findings provide descriptive evidence on health-literacy domains among younger adolescents in Umbria but should be interpreted cautiously given the low individual response rate, non-representative recruitment, use of a translated but not formally validated MOHLAA-Q in a younger age group, and exploratory associations. Further studies using age-appropriate, validated instruments and representative samples are warranted.

## 1 Introduction

Health literacy, defined as the ability to access, understand, evaluate, and use health information to make informed decisions about one’s health, is a fundamental skill throughout the lifespan (1). As highlighted by Nutbeam and colleagues, health literacy should not be viewed solely as an individual skill, but rather as a pivotal social determinant of health, influencing both personal well-being and population-level health (2). This multidimensional perspective has been further elaborated through integrative frameworks that underscore the importance of embedding health literacy within health systems and public health strategies, recognizing it as both an individual asset and a structural necessity for promoting equitable health outcomes (3, 4). International studies revealed that health literacy differs substantially across cultural and national contexts (5). While the evidence offers valuable insights into adult populations, the health literacy of adolescents remains underexplored. In fact, existing studies often focus on adults (6, 7), with limited evidence on adolescents and heterogeneity in the conceptualization and measurement of their health literacy. Available studies among adolescents have used different instruments and have addressed single dimensions of health literacy rather than providing a comprehensive assessment, with many focusing on its relationships with health behaviors and outcomes (8–12). These features contribute to an incomplete and heterogeneous evidence base on adolescent health literacy, particularly regarding its multidimensional assessment. Yet adolescence represents a critical developmental phase marked by profound physical, psychological, and social changes. In this period, the ability to effectively engage with health information is closely linked to healthier behaviors, improved disease management, and the adoption of sustainable lifestyle choices. Higher levels of health literacy in adolescence have been associated with more favorable health-related behaviors and outcomes (8, 13). Fostering health literacy during adolescence can thus enhance self-care capabilities and contribute to the promotion of lifelong health and well-being (13–16). Adolescence is also an important period for health promotion interventions that can be embedded in the school setting. In Italy, Schools Promoting Health exist that integrate physical, mental, and relational well-being into their daily organization and teaching programs, by promoting awareness and competencies with technical and scientific support from professionals of local health authorities. The impact of such initiatives can be monitored through population-based surveillance such as the Italian Health Behavior in School-aged Children (HBSC-Italy), that provides systematic information on adolescents’ health, behaviors, and social determinants. In the latest survey, conducted in 2022, all Italian Regions participated. HBSC also includes the Health Literacy for School-Aged Children scale (HLSAC) but its use within the Italian context is fragmented and limited to regional pilot experiences. Therefore, although HBSC addresses relevant determinants of health, including health and risk behaviors and socioeconomic factors, it does not provide an assessment of adolescent health literacy in Italy. Few studies have specifically investigated health literacy (17) and digital health literacy (18) among adolescents in Italy. Specifically, the study by Velasco et al. investigated the validity and reliability of the HLSAC. Although the HLSAC represents an important reference for research on adolescent health literacy in Italy, given the availability of a validated Italian version (17), further tools are available to measure it among adolescents, with different conceptual and measurement characteristics (19). Among them, the Measurement of Health Literacy Among Adolescents Questionnaire (MOHLAA-Q) is a multidimensional instrument covering different domains of health literacy, including dealing with health-related information, communication and interaction, attitudes toward health, and health-related knowledge, which was originally developed and validated in adolescents aged 14–17 years (20). We decided to use the MOHLAA-Q, even though an Italian version had not been previously formally validated, because its multidimensional structure was considered particularly suited to the objectives of our analysis, namely to describe health literacy through its different dimensions. Because the HLSAC and MOHLAA-Q differ in their conceptualization, item content, scoring, and domain structure, results obtained with the two instruments are not comparable and should be interpreted as complementary. The present manuscript reports a secondary analysis of data collected within a broader study primarily designed to investigate adolescents’ vaccination and its determinants. Within that broader study, the present secondary analysis aimed to describe four specific health-literacy domain among adolescents attending lower secondary schools in Umbria, Italy, using the MOHLAA-Q. A further objective of the present secondary analysis was to explore, as a hypothesis-generating analysis, the associations between each health-literacy domain and selected socio-demographic and family-related characteristics. These included adolescent sex, school province, responding-parent citizenship, educational attainment and occupation, parental health literacy, religion, and political orientation. The findings may contribute to informing health literacy promotion strategies.

## 2 Materials and methods

### 2.1 Study design

The present manuscript reports a secondary analysis of data collected within a broader cross-sectional study on adolescents’ vaccination and its determinants. The original study was conducted through an online survey in lower secondary schools of Umbria, a region of central Italy, from 23 April to 9 June 2024. The recruitment of schools was carried out with the support of the Regional School Office, which first contacted the principals of the 72 lower secondary schools in Umbria to introduce the project. The willingness to participate was subsequently collected by researchers through telephone contacts. Eighteen schools agreed to participate, corresponding to a school participation rate of 25.0% (18/72). Participating schools were then sent a note to be published on their electronic school register, including the link to the survey and the study information materials. Through participating schools, 6,996 eligible adolescents were reached overall. The study protocol was approved by the Umbria Regional Ethics Committee (protocol no. 26538/22/OV approved on 3 January 2023).

### 2.2 Study population

The request to complete the survey was addressed to students attending the third year of lower secondary schools and to their parents. Individual age was not collected as part of the questionnaire, and eligibility was therefore defined according to school grade rather than chronological age. Participants were eligible for inclusion if at least one parent was proficient in Italian, ensuring effective comprehension and communication. Finally, participation was contingent upon the provision of parental informed consent and adolescent assent, confirming voluntary agreement to take part in the research.

### 2.3 Sample size

No formal *a priori* sample-size calculation was performed for the present secondary analysis. The sample size was determined by the recruitment strategy of the broader study, which aimed to disseminate the questionnaire through all lower secondary schools in the Umbria Region. The final sample was therefore opportunistically determined by the willingness of schools to participate and by the willingness of adolescents and parents to complete the questionnaire. No assumptions regarding the expected prevalence of any outcome, desired precision, response rate, or design effect due to school-level clustering were used to determine the sample size for the original study.

### 2.4 Data collection

Data for the present secondary analysis were obtained from the questionnaire administered as part of the broader study on adolescents’ vaccination and its determinants. Data were collected through an online questionnaire, which gathered information on adolescents’ and responding parents’ health literacy and a range of adolescents’, responding parents’ and spouses/cohabitants’ individual, socio-demographic, and cultural characteristics. The questionnaire was administered through a single electronic link shared through the electronic school register on 23 April with two reminders sent in May. The questionnaire remained accessible up to 9 June. The responding parent was requested to first provide the consent before the adolescent could provide his/her assent and responses. Once the adolescent completed the section of the questionnaire dedicated to them, the responding parent then completed the final part of the questionnaire through the same survey link. Hence, the questionnaire embedded a predefined sequence of sections, with the responding parent’s consent and the adolescent’s assent first, followed by the adolescent section, including the MOHLAA-Q, and a last section aimed to collect responding parents’ and spouses/cohabitants’ information. Inclusion of questionnaire responses was not conditional on completion by both the adolescent and the responding parent; consequently, adolescents could complete the questionnaire without a corresponding parent questionnaire, but the other way around was not possible as MOHLAA-Q questions were mandatory. The use of the same survey link allowed adolescents’ and responding parents’ responses to be automatically linked without the need to collect direct personal identifiers. Unmatched adolescents’ records were retained for analyses of adolescents’ health literacy but were excluded from those requiring parent–adolescent linkage of information. The survey link was distributed exclusively through the electronic school register of participating schools and was therefore not openly accessible. The survey platform was configured to allow one response per account. Once the questionnaire had been completed by both the adolescent and the responding parent, the survey could not be accessed again to submit an additional response. This access restriction was tested before the beginning of data collection. IP-address or cookie-based duplicate checks were not used. Completion time was monitored as a quality-control measure, and no anomalously short or otherwise implausible completion times were recorded. The platform retained partially completed questionnaires. For assessing adolescents’ health literacy, the MOHLAA-Q was employed. This 29-item tool was originally developed and validated among individuals aged 14 to 17 years and uses a 4- or 5-point Likert scale and closed questions to assess four subdomains: (A) Difficulty in managing health-related information (cognitive and behavioral); (B) Health-related communication skills (behavioral-communicative); (C) Attitudes toward personal health and health information (self-awareness, self-control, motivation, and interest); (D) Health-related knowledge. The MOHLAA-Q was translated into Italian by the authors except, through a collaborative process. All authors participated in the translation and discussed each item collectively until consensus was reached on the final Italian wording. No modifications were made to the content of the items or to the response categories of the original questionnaire. Accordingly, the scoring procedures described in the original MOHLAA-Q development and validation study were retained. Responding parents’ health literacy was assessed using the Italian version of the Medical Term Recognition Test (IMETER), which has been previously validated in Italian by Biasio et al. (21). This one-page instrument includes two columns comprising 40 commonly used medical terms and 30 non-medical or fictitious terms. Following the Italian validation by Biasio et al. (21), one point was assigned for each correctly identified medical term, while one point was subtracted for each incorrectly identified non-medical or fictitious term, yielding an adjusted score. Through the questionnaire, socio-demographic and cultural information was also collected. Adolescent-level variables included the province of the attended school and sex. Responding parent-level variables included kinship, citizenship, geographical origin (North America, South America, Europe, Africa, Asia, Oceania, and other), religious orientation, political orientation, educational attainment, and occupation. Educational attainment and occupation of the spouse/cohabitant were also collected through the responding parent. Socio-demographic and cultural questions were mandatory, with the exception of sex and political orientation, which were optional. Religious orientation was categorized as Catholic Christian, Orthodox Christian, Muslim, Jewish, Protestant Christian, Buddhist, Hindu, prefer not to answer, or other (specify). Educational attainment was categorized as none, primary school, lower secondary school, upper secondary school, university degree, or postgraduate degree. Occupation was categorized as full-time employee, part-time employee, self-employed/professional, homemaker, seeking employment, unemployed, or other (specify). Political orientation was assessed using a 0–10 scale ranging from strongly left-wing (0) to strongly right-wing (10). Scores were categorized as left-wing political orientation (0–3), centrist political orientation (4–6), or right-wing political orientation (7–10). The complete Italian versions of the adolescent and responding parent questionnaires, including item wording, response options, scoring procedures, and coding rules, are provided as Supplementary Material 1.

### 2.5 Psychometric evaluation of the Italian MOHLAA-Q

The psychometric performance of the Italian MOHLAA-Q was evaluated before examining associations with participant characteristics. Item completion was assessed for all 29 items by calculating the number and percentage of valid and missing responses. The proportion of participants with complete responses to all items was also determined. Internal consistency was evaluated using Cronbach’s alpha for Scales A–C. For Scale D, which consists of dichotomous knowledge items scored as 0 for an incorrect response and 1 for a correct response, internal consistency was assessed using the Kuder–Richardson Formula 20 coefficient. Accordingly, the internal consistency and factor-analytic findings for Scale D were considered informative about the coherence of its items and the plausibility of a unidimensional latent interpretation, but not as the basis for judging the usefulness of the predefined knowledge score. Item performance was examined using corrected item-total correlations, calculated by correlating each item with the sum of the remaining items in the corresponding scale. Correlations below 0.30 were considered indicative of potentially weak item performance, while negative correlations prompted verification of item coding and conceptual coherence. For Scale D, item difficulty was calculated as the proportion of correct responses, and corrected point-biserial correlations were used to examine item discrimination. A correlated four-factor model corresponding to the four MOHLAA-Q scales was evaluated using confirmatory factor analysis. For Scale D, this analysis was considered exploratory and was used to examine the extent to which the eight knowledge items supported a common latent dimension; given the content-specific nature of the knowledge questions, lack of strong factor loadings was not interpreted as evidence that the predefined knowledge score itself was invalid. Because the questionnaire items were ordinal or dichotomous, models were estimated using the weighted least squares mean- and variance-adjusted estimator. Model fit was evaluated using the comparative fit index, Tucker–Lewis index, root mean square error of approximation with its 95% confidence interval, and standardized root mean square residual. Comparative fit index and Tucker–Lewis index values of at least 0.90 and root mean square error and standardized root mean square residual values of no more than 0.08 were considered indicative of approximately acceptable fit. These thresholds were treated as descriptive guidelines rather than strict decision rules. Standardized factor loadings were also examined, with loadings below 0.30 flagged as potentially weak. Missing item responses in the confirmatory factor analysis were handled using pairwise-present information under the weighted least squares estimator. These analyses were considered sample-specific and exploratory and were not intended to constitute a formal validation of the Italian version of the MOHLAA-Q. In particular, the psychometric findings for Scale D were not interpreted as evidence supporting a homogeneous latent health-knowledge construct, but rather as information on the internal coherence of a content-diverse knowledge score.

### 2.6 Statistical analysis

Descriptive statistics were used to summarize data. For describing the responses to the MOHLAA-Q, in accordance with the scientific literature, mean scores were calculated for Scales A, B, and C, while a total score was computed for Scale D (20). All 29 MOHLAA-Q items were mandatory in the online questionnaire; therefore, questionnaires with interrupted completion were excluded. Consequently, there were no item-level missing data on MOHLAA-Q. Adolescents’ health literacy was classified as follows: for the MOHLAA-Q scale A, mean scores were categorized as follows: 1–2.5, many difficulties; > 2.5–3, some difficulties; > 3–3.5, few difficulties; > 3.5–4, barely/no difficulties. For scale B the following classification was considered: 1–2.5, low communication/interaction skills; > 2.5–3, moderate skills; > 3–3.5: rather high skills; > 3.5–4: high skills. For scale C the following criteria were adopted: 1–3: passive attitudes; > 3–4: partially passive/active attitudes, > 4–5: active attitudes. For scale D a score from 0 to 3 was considered representing low health-related knowledge, 4–5 moderate knowledge and 6–8 high knowledge. For Scale D, these categories were based on the original MOHLAA-Q scoring scheme and were interpreted as ordered levels of the health-related knowledge score, based on questions covering different content areas, rather than as levels of a validated unidimensional latent trait. These original MOHLAA-Q thresholds were developed and validated in adolescents aged 14–17 years and were applied in the present study, although the chronological age of individual participants was not collected and the study population was defined according to school grade. In contrast, few socio-demographic and cultural variables were not mandatory and therefore some predictor data were missing. For the exploratory analyses, a complete-case approach was applied separately to each predictor–outcome analysis; observations with missing data for the predictor under consideration were excluded from that specific analysis. Consequently, the analytical denominator varied according to the availability of the predictor data, while the MOHLAA-Q outcomes were based on complete item-level data. Responding parents’ health literacy was classified according to the thresholds reported in the Italian validation of the IMETER by Biasio et al. (21): low (0–20), marginal (21–34), and functional (35–40) health literacy. All IMETER items were mandatory; therefore, the responding parents who completed the parent questionnaire provided complete IMETER responses, and no questionnaires were excluded on the basis of incomplete IMETER data. Negative adjusted scores were potentially possible and included in the lowest category (≤20) in accordance with the applied classification rule. Prior to conducting the single-predictor (univariable) proportional-odds ordinal logistic regression models, selected socio-demographic variables were recoded for analytical purposes. Educational attainment was dichotomized into “up to high school” (including the responses “None,” “Middle school,” and “High school”) and “degree or more” (including “Degree” and “Post-graduate”). Employment status was classified into three categories: Employed, Unemployed, and Househusband/Housewife. Citizenship was recoded as Italian and non-Italian and religious orientation was categorized as follows: Catholic Christian, Orthodox Christian, Atheist, and other religions/not responding. Single-predictor (univariable) proportional-odds ordinal logistic regression models were fitted to explore associations between socio-demographic and cultural characteristics and the four MOHLAA-Q domains. The original ordinal categories of each MOHLAA-Q scale were retained for the primary analyses, and no dichotomization of the MOHLAA-Q outcomes was performed. For Scale A, categories were ordered from “many difficulties” to “barely/no difficulties,” so that higher categories represented fewer difficulties in dealing with health-related information. For Scale B, categories were ordered from “low” to “high” communication and interaction skills; for Scale C, from “passive” to “active” attitudes; and for Scale D, from “low” to “high” health-related knowledge score. Associations between each predictor and each MOHLAA-Q domain were examined separately using proportional-odds ordinal logistic regression models. The ordinal odds ratio (OR) represents the odds of being in a higher outcome category. For Scale D, a higher outcome category was interpreted as a higher category of the predefined health-related knowledge score, rather than as evidence of a higher level of a homogeneous latent health-knowledge construct. The primary models used HC1 robust standard errors clustered by school to account for the clustering of observations within schools. The proportional-odds assumption was evaluated for each fitted model using a cluster-robust Brant-type Wald test based on separate cumulative binary logistic regressions, retaining the same complete-case sample, eligible predictor categories, and school-level clustering as the corresponding primary analysis. A *p*-value below 0.05 was considered evidence against the parallel-lines assumption. When the assumption was violated, threshold-specific cumulative ORs with school-clustered 95% confidence intervals were reported. Diagnostics affected by non-convergence or complete or quasi-complete separation were classified as indeterminate. The first eligible predictor category was used as the reference category. Categories with fewer than 10 complete observations were retained for descriptive purposes but were excluded from ordinal regression because of sparse data; the corresponding OR was therefore reported as not estimable. Pearson’s chi-square or Fisher’s exact tests were also reported as secondary unadjusted categorical analyses. These tests did not account for the ordinal nature of the MOHLAA-Q outcomes or for clustering by school. No *a priori* hierarchical testing strategy was specified; therefore, *p*-values were interpreted as nominal and exploratory, taking into account the number of comparisons performed. Statistical analyses were conducted using StataNow (22). A *p* value ≤ 0.05 was considered statistically significant. Given the exploratory nature of these analyses, associations between the four MOHLAA-Q domains and socio-demographic and cultural characteristics were interpreted as exploratory.

## 3 Results

A total of 340 adolescents provided complete answers to the MOHLAA-Q, corresponding to an individual completion rate of 4.9% (340/6,996). Overall, 505 adolescents accessed the online questionnaire, of whom 2 did not provide assent, while 503 provided assent (99.6%). Of those providing assent, 340/503 (67.6%) completed the mandatory MOHLAA-Q and were included in the adolescent analytical sample. For the parent component, 602 parents initiated the questionnaire by accessing the survey link and 565/602 (93.9%) provided consent. Of those providing consent, 277/565 (49.0%) completed the parent questionnaire and were successfully linked to the corresponding adolescent questionnaire, resulting in 277 adolescent–parent pairs. Thus, the parent questionnaire was available and successfully linked for 81.5% (277/340) of the adolescents included in the MOHLAA-Q analytical sample. These 277 linked pairs constituted the analytic sample for analyses involving parental characteristics. Analyses based exclusively on adolescent-level variables included all 340 adolescents who completed the MOHLAA-Q. The figures referring to school- and individual-level participation are reported in the study flowchart (Figure 1). No individual-level information was available for adolescents who did not respond to the questionnaire, and no sufficiently detailed comparative characteristics were available for the 54 non-participating schools. Therefore, comparisons between participating and non-participating schools and between responders and non-responders could not be performed (Table 1).

**Figure 1:**
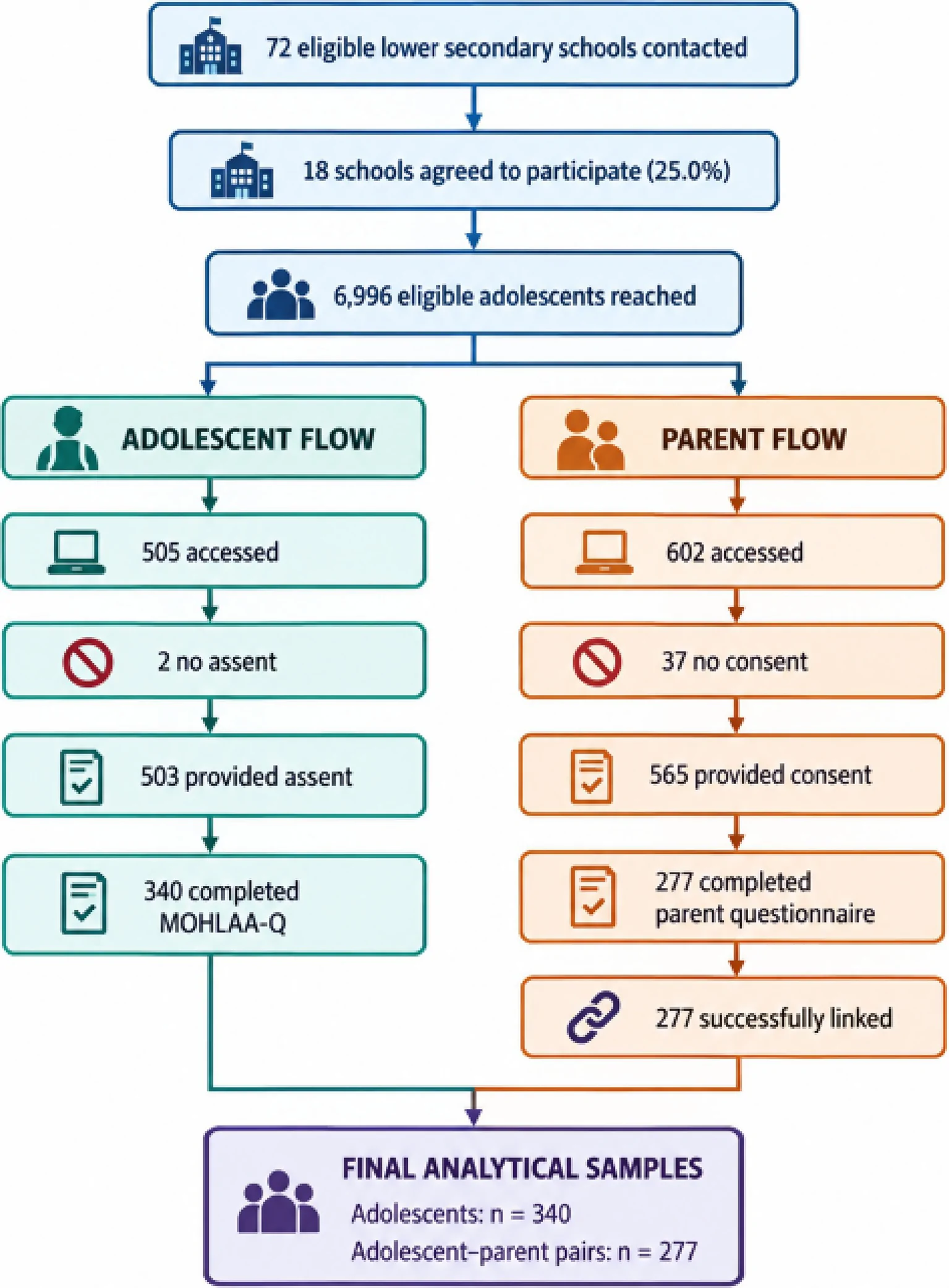
Study flowchart.

**Table 1:** Socio-demographic characteristics of the sample.

Socio-demographic variable	Frequency (N)	Percentage (%)
Adolescents		
Sex (293)		
Female	154	52.6
Male	139	47.4
Province (340)		
Perugia	298	87.6
Terni	42	12.4
Responding parents (277)		
Mother	254	91.7
Father	23	8.3
Responding parents’ educational attainment (277)		
High school	138	49.8
Degree	87	31.4
Postgraduate	31	11.2
Middle school	20	7.2
None	1	0.4
Spouses/cohabitants’ educational attainment (277)		
High school	162	58.4
Middle school	50	18.1
Degree	48	17.3
Postgraduate	10	3.6
Not applicable	4	1. 4
Elementary school	2	0.7
None	1	0.4
Responding parents’ employment status (277)		
Househusband	21	7.6
Full-time employee	133	48
Part-time employee	78	28.1
Unemployed	2	0.7
Job seekers	2	0.7
Freelancer/Self-employed	41	14.8
Spouses/cohabitants’ employment status (277)		
Househusband	3	1.1
Full-time employee	178	64.3
Part-time employee	14	5.1
Unemployed	5	1.8
Freelancer/Self-employed	68	24.5
Not applicable	9	3.2
Responding parents’ geographical origin (by continent) (277)		
Europe	276	99.6
Asia	1	0.4
Responding parents’ citizenship (277)		
Italian	268	96.7
Romanian	4	1.4
Czech	2	0.7
Albanian	1	0.4
Filipino	1	0.4
German	1	0.4
Responding parents’ religious orientation (277)		
Atheist	25	9
Catholic Christian	234	84.5
Orthodox Christian	2	0.7
Muslim	1	0.4
I prefer not to answer	15	5.4
Responding parents’ political orientation (Optional) (147)		
Left-wing political orientation	55	37.4
Centrist political orientation	57	38.8
Right-wing political orientation	35	23.8

Among the adolescents considered for the analyses, 87.6% were from the Province of Perugia, while 12.4% were from the Province of Terni; sex was available for 293 participants (86.2%); among these, 52.6% were female and 47.4% were male. As for responding parents, the majority were mothers (91.7%) and Italian citizens (96.7%), with a marginal representation of individuals from Romania (1.4%), the Czech Republic (0.7%), Albania, the Philippines, and Germany (each 0.4%). Regarding geographic origin, almost all responding parents were of European origin (99.6%), while 0.4% were of Asian origin. Regarding religious orientation, the majority identified as Catholic Christians (84.5%). A smaller portion identified as atheists (9%), followed by Orthodox Christians (0.7%) and Muslims (0.4%). Additionally, 5.4% of participants preferred not to answer regarding their religious orientation. Regarding political orientation, among the 147 parents who provided a response, 37.4% reported a left-wing political orientation, 38.8% a centrist political orientation, and 23.8% a right-wing political orientation. Concerning responding parents’ educational attainment, nearly half (49.8%) reported having completed high school, while 31.4% held a university degree and 11.2% a postgraduate qualification. A smaller proportion had completed only middle school (7.2%), and a minimal percentage reported having no formal education (0.4%). In terms of employment status, 48% of responding parents were employed full-time, 28.1% were employed part-time, and 14.8% were self-employed or freelancers. Only a small minority were unemployed (0.7%) or seeking employment (0.7%), while 7.6% identified as househusbands. The educational attainment of spouses or cohabitants was as follows: 58.4% held a high school diploma, 17.3% had a university degree, and 3.6% a postgraduate degree. Additionally, 18.1% had completed middle school, while only a few had an elementary-level education (0.7%) or no qualifications at all (0.4%). Among spouses or cohabitants, the most common employment status was full-time employment (64.3%), followed by self-employment or freelance work (24.5%) and part-time employment (5.1%). A minority were unemployed (1.8%) or househusbands (1.1%).

### 3.1 Adolescents’ health literacy

Based on the 340 complete responses received (Table 2), the most frequently reported category for Scale A was “some difficulties” in dealing with health-related information (53.3%), followed by “few difficulties” (25.3%). A further 17.6% reported “many difficulties,” while 3.8% reported “barely/no difficulties.” For scale B, which measures communication and interactive skills, a rather balanced distribution was observed: 32.1% of adolescents reported a “high” level of communication skills, 31.2% a “rather high” level, 28.5% indicated a “moderate” level and only 8.2% reported a “low” level. Regarding scale C, related to attitudes towards one’s health and health information, more than half of the sample (51.2%) showed an “active” attitude, while 45.9% were in an intermediate position (“partly passive–partly active”). Only a small part of the sample (2.9%) showed a “passive” attitude. For scale D, which assesses health-related knowledge, the results revealed that 51.5% of adolescents had a “low” level of knowledge, while 40.9% had a “moderate” level. Only 7.6% of the sample said they had a “high” level of health knowledge.

**Table 2:** Adolescents’ health literacy according to MOHLAA-Q (*N* = 340).

	N (Frequency)	%	95% CI
Scale A (Dealing with health-related information)			
Many difficulties	60	17.6	13.7–22.1
Some difficulties	181	53.3	47.7–58.6
Few difficulties	86	25.3	20.7–30.2
Barely/No difficulties	13	3.8	2.1–6.4
Scale B (Communication and interaction skills)			
Low	28	8.2	5.5–11.7
Moderate	97	28.5	23.8–33.6
Rather High	106	31.2	26.3–36.4
High	109	32.1	27.1–37.3
Scale C (Attitudes toward one’s own health and health information)			
Passive	10	2.9	1.4–5.3
Partly Passive–Partly Active	156	45.9	40.5–51.3
Active	174	51.2	45.7–56.6
Scale D (Health-related knowledge)			
Low	175	51.5	46.1–56.9
Moderate	139	40.9	35.6–46.3
High	26	7.6	5.1–11.1

### 3.2 Psychometric properties of the MOHLAA-Q

Complete item-level data were available for all 340 participants. No missing responses were observed across the 29 MOHLAA-Q items. Scale A showed acceptable internal consistency (Cronbach’s alpha = 0.790), with corrected item-total correlations ranging from 0.354 to 0.540. Scale B showed lower internal consistency (Cronbach’s alpha = 0.593), although all corrected item-total correlations were positive and ranged from 0.350 to 0.465. Scale C showed low internal consistency (Cronbach’s alpha = 0.533); corrected item-total correlations ranged from 0.095 to 0.445, with two of the five items showing values below 0.30. Scale D also showed low internal consistency (KR-20 = 0.199; Table 3). Detailed item-level descriptive and discrimination statistics are reported in Supplementary Material II, Table S2. The correlated four-factor confirmatory model converged. Evidence of model fit was mixed. RMSEA indicated acceptable approximate fit (0.047; 95% CI: 0.041–0.053), whereas CFI (0.888), TLI (0.877), and SRMR (0.081) did not uniformly meet the predefined approximate criteria. Standardized factor loadings were positive for all items in Scales A and B, ranging from 0.475 to 0.742 and from 0.432 to 0.683, respectively. For Scale C, standardized loadings ranged from 0.213 to 0.718, with one of the five items showing a loading below 0.30. Detailed model-fit indices and standardized factor loadings are reported in Supplementary Material 2, Table S3. For the dichotomous knowledge items of Scale D, item difficulty, expressed as the proportion of correct responses, ranged from 0.076 to 0.656. Corrected point-biserial correlations ranged from −0.038 to 0.126, indicating limited item discrimination. Standardized factor loadings were also generally weak, ranging from −0.111 to 0.344, with seven of the eight items showing loadings below 0.30. Taken together, these findings provide limited evidence of internal coherence and unidimensionality for Scale D in the present sample. Accordingly, Scale D was retained as a predefined ordered measure of health-related knowledge and used for descriptive and exploratory association analyses, but interpreted cautiously, without assuming that it represents a homogeneous latent construct.

**Table 3:** Psychometric properties of the Italian MOHLAA-Q.

Scale	N. Items	Internal consistency	Corrected item-total correlation	Standardized loading range
A	12	α = 0.790	0.354–0.540	0.475–0.742
B	4	α = 0.593	0.350–0.465	0.432–0.683
C	5	α = 0.533	0.095–0.445	0.213–0.718
D	8	KR-20 = 0.199	−0.038–0.126	−0.111–0.344

α, Cronbach’s alpha; KR-20, Kuder–Richardson Formula 20. Corrected item-total correlations for Scale D are corrected point-biserial correlations. All analyses were based on *N* = 340 participants; no missing item responses were observed.

### 3.3 Analysis of differences in health literacy according to socio-demographic and cultural characteristics

Univariate associations between socio-demographic and cultural characteristics and the four dimensions of health literacy were examined using proportional-odds ordinal logistic regression, retaining the original ordered categories of each MOHLAA-Q scale. Each predictor was analyzed separately, with HC1 robust standard errors clustered by school. The school-clustered overall *p*-value from the omnibus Wald test was considered the primary test of association. Of the 44 scale–predictor combinations, ordinal regression could not be estimated for the four associations involving responding parents’ citizenship. Among the 40 fitted logistic regression models, proportional-odds diagnostics were reliable for 35 models: 33 showed no evidence against the assumption, while two showed evidence of non-proportionality. They were those assessing the association between sex and Scale A (χ^2^ = 6.72, df = 2, *p* = 0.035) and sex and Scale B (χ^2^ = 8.06, df = 2, *p* = 0.018). Threshold-specific estimates were therefore examined for these associations. The diagnostic was indeterminate for the remaining five models: employment status with Scales A and C, and political beliefs with Scales A, C, and D. Sparse or empty predictor-by-outcome cells resulted in complete or quasi-complete separation and non-convergence. Complete diagnostic results and threshold-specific estimates are reported in Supplementary Material 2, Table S4. For Scale A (dealing with health-related information; Table 4), significant associations were observed with adolescents’ sex and with the educational attainment of the responding parent’s spouse/cohabitant. However, the proportional-odds assumption was violated for the association between sex and Scale A (χ^2^ = 6.72, df = 2, *p* = 0.035); therefore, a single common ordinal OR was not considered an adequate summary of this association. In the threshold-specific analyses, compared with female adolescents, males had higher odds of being above the “many difficulties” (OR 3.68, 95% CI 2.00–6.78), above the “some difficulties” category (OR 1.66, 95% CI 1.02–2.70), and above the “few difficulties” category (OR 4.03, 95% CI 1.02–15.96) rather than any lower category. Thus, male adolescents were generally more likely to report fewer difficulties, although the magnitude of the association varied across outcome thresholds. Similarly, adolescents whose respondent’s spouse/cohabitant had a university degree or higher qualification had higher odds of being in a higher Scale A category than those whose spouse/cohabitant had lower educational attainment (OR 1.55, 95% CI 1.00–2.40; *p* = 0.048). No statistically significant associations were observed for the other examined characteristics. For Scale B (communication and interaction skills; Table 5), the respondent’s employment status was significantly associated with the ordinal outcome (overall *p* = 0.019). Compared with adolescents whose responding parent was employed, those whose responding parent was a househusband had higher odds of being in a higher category of communication and interaction skills (OR 3.19, 95% CI 1.22–8.36; *p* = 0.019). The proportional-odds assumption was also violated for the association between sex and Scale B (χ^2^ = 8.06, df = 2, *p* = 0.018). The threshold-specific male-versus-female ORs were 1.12 (95% CI 0.58–2.18) for being above the “low” category, 1.14 (95% CI 0.71–1.84) for being above the “moderate” category, and 2.28 (95% CI 1.27–4.09) for being in the “high” category rather than any lower category. This finding suggests that the association with sex was concentrated at the highest Scale B threshold. No significant associations were observed for the other characteristics examined. For Scale C (attitudes toward one’s own health and health information; Table 6), no significant associations were observed with the examined socio-demographic and cultural characteristics. For Scale D (health-related knowledge; Table 7), responding parents’ health literacy, as assessed by the IMETER, was significantly associated with the ordinal outcome (overall *p* = 0.017). Compared with adolescents whose responding parent had low health literacy, those whose parent had marginal health literacy had higher odds of being in a higher category of health-related knowledge (OR 1.84, 95% CI 1.07–3.16; *p* = 0.029), while the corresponding odds were also higher among those whose parent had functional health literacy (OR 3.57, 95% CI 1.49–8.56; *p* = 0.005). No statistically significant associations were observed between Scale D and the other examined socio-demographic and cultural characteristics.

**Table 4:** Scale A (Dealing with health-related information).

Characteristic	Level	Many	Some	Few	Barely/No	Ordinal OR (95% CI)	OR *p*-value	School-clustered overall p-value	Secondary categorical *p*-value
Adolescents									
Sex (293) †	Female	37 (24.0%)	79 (51.3%)	36 (23.4%)	2 (1.3%)	Reference	–	–	<0.001‡
	Male	11 (7.9%)	79 (56.8%)	42 (30.2%)	7 (5.0%)	Some/Few/Barely-No vs. Many: 3.68 (2.00–6.78)			
						Few/Barely-No vs. Many/Some: 1.66 (1.02–2.70)			
						Barely/No vs. Many/Some/Few: 4.03 (1.02–15.96)			
Province (340)	Terni	6 (14.3%)	21 (50.0%)	14 (33.3%)	1 (2.4%)	–	0.176	0.176	0.663‡
	Perugia	54 (18.1%)	160 (53.7%)	72 (24.2%)	12 (4.0%)	0.75 (0.49–1.15)			
Responding parents (277)									
Parentage	Mother	42 (16.5%)	136 (53.5%)	69 (27.2%)	7 (2.8%)	–	0.764	0.764	0.800‡
	Father	4 (17.4%)	13 (56.5%)	5 (21.7%)	1 (4.3%)	0.89 (0.40–1.96)			
Responding parents’ educational attainment	Up to high school	29 (18.2%)	81 (50.9%)	46 (28.9%)	3 (1.9%)	–	0.885	0.885	0.382‡
	University degree or over	17 (14.4%)	68 (57.6%)	28 (23.7%)	5 (4.2%)	1.04 (0.60–1.82)			
Spouses/cohabitants’ educational attainment	Up to high school	40 (18.3%)	117 (53.4%)	58 (26.5%)	4 (1.8%)	–	0.048	0.048	0.126‡
	University degree or over	6 (10.3%)	32 (55.2%)	16 (27.6%)	4 (6.9%)	1.55 (1.00–2.40)			
Responding parents’ employment status §	Employed	44 (17.5%)	137 (54.4%)	63 (25.0%)	8 (3.2%)	–	0.090	0.090	0.529‡
	Unemployed	0 (0.0%)	2 (50.0%)	2 (50.0%)	0 (0.0%)	NE			
	Househusband	2 (9.5%)	10 (47.6%)	9 (42.9%)	0 (0.0%)	1.78 (0.91–3.49)			
Spouses/cohabitants’ employment status	Employed	44 (16.9%)	139 (53.5%)	70 (26.9%)	7 (2.7%)	–	0.642	0.642	0.535‡
	Unemployed	1 (7.1%)	9 (64.3%)	3 (21.4%)	1 (7.1%)	1.29 (0.43–3.94)			
	Househusband	1 (33.3%)	1 (33.3%)	1 (33.3%)	0 (0.0%)	NE			
Responding parents’ citizenship	Italian	45 (16.8%)	144 (53.7%)	71 (26.5%)	8 (3.0%)	NE	–		1.000‡
	Not Italian	1 (11.1%)	5 (55.6%)	3 (33.3%)	0 (0.0%)	NE			
Responding parents’ religious orientation	Catholic Christian	40 (17.1%)	130 (55.6%)	59 (25.2%)	5 (2.1%)	–	–	0.185	0.284‡
	Orthodox Christian	0 (0.0%)	1 (50.0%)	1 (50.0%)	0 (0.0%)	NE	–		
	Atheist	3 (12.0%)	11 (44.0%)	10 (40.0%)	1 (4.0%)	1.91 (0.95–3.84)	0.068		
	Other religions/not responding	3 (18.8%)	7 (43.8%)	4 (25.0%)	2 (12.5%)	1.50 (0.39–5.85)	0.550		
Responding parents’ Political orientation (Optional) (147) §	Left-wing political orientation	9 (16.4%)	24 (43.6%)	22 (40.0%)	0 (0.0%)	–	–	0.319	0.192‡
	Centrist political orientation	11 (19.3%)	33 (57.9%)	13 (22.8%)	0 (0.0%)	0.58 (0.28–1.21)	0.141		
	Right-wing political orientation	9 (25.7%)	14 (40.0%)	11 (31.4%)	1 (2.9%)	0.69 (0.25–1.93)	0.474		
Parental health literacy–IMETER	Low literacy	10 (15.9%)	38 (60.3%)	14 (22.2%)	1 (1.6%)	–	–	0.328	0.431‡
	Marginal literacy (limited)	23 (13.9%)	89 (53.9%)	47 (28.5%)	6 (3.6%)	1.37 (0.73–2.56)	0.323		
	Functional literacy (adequate)	13 (26.5%)	22 (44.9%)	13 (26.5%)	1 (2.0%)	0.85 (0.33–2.19)	0.738		

The ordinal OR estimates the odds of being in a higher judgment category. The first eligible predictor category is the reference. OR confidence intervals, OR *p*-values and the omnibus Wald *p*-value use HC1 robust standard errors clustered by school (R), with t/F reference distributions based on G − 1 degrees of freedom. The school-clustered overall *p*-value is the primary omnibus Wald F test. The secondary categorical *p*-value uses Pearson’s chi-square test when every expected count is ≥5, Fisher’s exact test for sparse 2 × 2 tables (†), or Fisher’s test with Monte Carlo simulation for sparse R × C tables (‡; B = 100,000). The secondary test ignores outcome ordering and school clustering. Categories with fewer than 10 complete observations are shown descriptively but excluded from ordinal regression; their OR is reported as NE. NE, not estimable. CI, confidence interval; OR, odds ratio. † The proportional-odds assumption was violated for the association between sex and Scale A (cluster-robust Brant-type Wald test: χ^2^ = 6.72, df = 2, *p* = 0.035). Therefore, threshold-specific cumulative odds ratios are reported instead of a common proportional odds ratio. These compare Some/Few/Barely-No versus Many, Few/Barely-No versus Many/Some, and Barely-No versus Many/Some/Few difficulties, respectively, rather than a single common odds ratio. At each threshold, the odds ratio compares male with female adolescents; a value greater than 1 indicates higher odds among males of being above that threshold. Confidence intervals and *p*-values are based on HC1 robust standard errors clustered by school. § The proportional-odds diagnostic was indeterminate because empty or sparse predictor-by-outcome cells resulted in complete or quasi-complete separation and non-convergence of at least one diagnostic cumulative binary model. The proportional-odds estimates should therefore be interpreted cautiously.

**Table 5:** Scale B (Communication and interaction skills).

Characteristic	Level	Low	Moderate	Rather high	High	Ordinal OR (95% CI)	OR *p*-value	School-clustered overall *p-*value	Secondary categorical *p*-value
Adolescents									
Sex (293) †	Female	14 (9.1%)	42 (27.3%)	54 (35.1%)	44 (28.6%)	Reference	—	—	0.249
	Male	11 (7.9%)	37 (26.6%)	37 (26.6%)	54 (38.8%)	High/Rather high/Moderate vs. Low: 1.12 (0.58–2.18)	0.733		
						High/Rather high vs. Low/Moderate: 1.14 (0.71–1.84)	0.579		
						High vs. Low/Moderate/Rather high: 2.28 (1.27–4.09)	0.007		
Province (340)	Terni	3 (7.1%)	13 (31.0%)	11 (26.2%)	15 (35.7%)	–	0.799	0.799	0.880‡
	Perugia	25 (8.4%)	84 (28.2%)	95 (31.9%)	94 (31.5%)	0.93 (0.52–1.66)			
Responding Parents (277)									
Parentage	Mother	22 (8.7%)	69 (27.2%)	79 (31.1%)	84 (33.1%)	–	0.986	0.986	0.682‡
	Father	1 (4.3%)	6 (26.1%)	10 (43.5%)	6 (26.1%)	1.01 (0.49–2.07)			
Responding parents’ educational attainment	Up to high school	13 (8.2%)	40 (25.2%)	50 (31.4%)	56 (35.2%)	–	0.317	0.317	0.700
	University degree or over	10 (8.5%)	35 (29.7%)	39 (33.1%)	34 (28.8%)	0.79 (0.50–1.26)			
Spouses/cohabitants’ educational attainment	Up to high school	19 (8.7%)	60 (27.4%)	69 (31.5%)	71 (32.4%)	–	0.722	0.722	0.970‡
	University degree or over	4 (6.9%)	15 (25.9%)	20 (34.5%)	19 (32.8%)	1.10 (0.66–1.83)			
Responding parents’ employment status	Employed	23 (9.1%)	70 (27.8%)	82 (32.5%)	77 (30.6%)	–	–	0.019	0.191‡
	Unemployed	0 (0.0%)	2 (50.0%)	1 (25.0%)	1 (25.0%)	NE			
	Househusband	0 (0.0%)	3 (14.3%)	6 (28.6%)	12 (57.1%)	3.19 (1.22–8.36)	0.019		
Spouses/cohabitants’ employment status	Employed	20 (7.7%)	69 (26.5%)	85 (32.7%)	86 (33.1%)	–	0.377	0.377	0.394‡
	Unemployed	2 (14.3%)	5 (35.7%)	3 (21.4%)	4 (28.6%)	0.60 (0.19–1.91)			
	Househusband	1 (33.3%)	1 (33.3%)	1 (33.3%)	0 (0.0%)	NE			
Responding parents’ citizenship §	Italian	22 (8.2%)	74 (27.6%)	84 (31.3%)	88 (32.8%)	NE	–		0.396‡
	Not Italian	1 (11.1%)	1 (11.1%)	5 (55.6%)	2 (22.2%)	NE			
Responding parents’ religious orientation	Catholic Christian	19 (8.1%)	60 (25.6%)	78 (33.3%)	77 (32.9%)	–	–	0.710	0.705‡
	Orthodox Christian	0 (0.0%)	2 (100.0%)	0 (0.0%)	0 (0.0%)	NE	–		
	Atheist	2 (8.0%)	7 (28.0%)	8 (32.0%)	8 (32.0%)	0.94 (0.45–1.95)	0.866		
	Other religions/not responding	2 (12.5%)	6 (37.5%)	3 (18.8%)	5 (31.2%)	0.65 (0.22–1.94)	0.431		
Responding parents’ political orientation (Optional) (147)	Left-wing political orientation	7 (12.7%)	20 (36.4%)	12 (21.8%)	16 (29.1%)	–	–	0.552	0.286‡
	Centrist political orientation	3 (5.3%)	16 (28.1%)	25 (43.9%)	13 (22.8%)	1.37 (0.70–2.69)	0.349		
	Right-wing political orientation	4 (11.4%)	12 (34.3%)	10 (28.6%)	9 (25.7%)	1.03 (0.45–2.37)	0.935		
Parental health literacy–IMETER	Low literacy	6 (9.5%)	16 (25.4%)	16 (25.4%)	25 (39.7%)	–	–	0.700	0.688‡
	Marginal literacy (limited)	13 (7.9%)	45 (27.3%)	54 (32.7%)	53 (32.1%)	0.86 (0.42–1.73)	0.656		
	Functional literacy (adequate)	4 (8.2%)	14 (28.6%)	19 (38.8%)	12 (24.5%)	0.71 (0.31–1.65)	0.418		

The ordinal OR estimates the odds of being in a higher judgment category. The first eligible predictor category is the reference. OR confidence intervals, OR *p*-values and the omnibus Wald *p*-value use HC1 robust standard errors clustered by school (R), with t/F reference distributions based on G − 1 degrees of freedom. The school-clustered overall *p*-value is the primary omnibus Wald F test. The secondary categorical *p*-value uses Pearson’s chi-square test when every expected count is ≥5, Fisher’s exact test for sparse 2 × 2 tables (†), or Fisher’s test with Monte Carlo simulation for sparse R × C tables (‡; B = 100,000). The secondary test ignores outcome ordering and school clustering. Categories with fewer than 10 complete observations are shown descriptively but excluded from ordinal regression; their OR is reported as NE. NE, not estimable. CI, confidence interval; OR, odds ratio. † The proportional-odds assumption was violated for the association between sex and Scale B (cluster-robust Brant-type Wald test: χ^2^ = 8.06, df = 2, *p* = 0.018). Therefore, threshold-specific cumulative odds ratios are reported instead of a common proportional odds ratio. These compare Moderate/Rather high/High versus Low, Rather high/High versus Low/Moderate, and High versus Low/Moderate/Rather high communication and interaction skills. At each threshold, the odds ratio compares male with female adolescents; a value greater than 1 indicates higher odds among males of being above that threshold. Confidence intervals and p-values are based on HC1 robust standard errors clustered by school. § Responding-parent citizenship was not included in the ordinal regression because fewer than two eligible predictor categories remained after applying the prespecified exclusion of categories with fewer than 10 complete observations. Consequently, the proportional-odds assumption was not assessed for this predictor.

**Table 6:** Scale C (Attitudes toward one’s own health and health information).

Characteristic	Level	Passive	Partly passive–Partly active	Active	Ordinal OR (95% CI)	OR *p*-value	School-clustered overall *p*-value	Secondary categorical *p*-value
Adolescents								
Sex (293)	Female	5 (3.2%)	71 (46.1%)	78 (50.6%)	–	–	0.443	0.799‡
	Male	4 (2.9%)	59 (42.4%)	76 (54.7%)	1.17 (0.77–1.78)	0.443		
Province (340)	Terni	2 (4.8%)	19 (45.2%)	21 (50.0%)	–	–	0.743	0.673‡
	Perugia	8 (2.7%)	137 (46.0%)	153 (51.3%)	1.10 (0.63–1.92)	0.743		
Responding parents (277)								
Parentage	Mother	9 (3.5%)	118 (46.5%)	127 (50.0%)	–	–	0.118	0.394‡
	Father	0 (0.0%)	8 (34.8%)	15 (65.2%)	1.93 (0.84–4.45)	0.118		
Responding parents’ educational attainment	Up to high school	4 (2.5%)	79 (49.7%)	76 (47.8%)	–	–	0.344	0.215‡
	University degree or over	5 (4.2%)	47 (39.8%)	66 (55.9%)	1.32 (0.74–2.36)	0.344		
Spouses/cohabitants’ educational attainment	Up to high school	8 (3.7%)	100 (45.7%)	111 (50.7%)	–	–	0.675	0.861‡
	University degree or over	1 (1.7%)	26 (44.8%)	31 (53.4%)	1.15 (0.59–2.25)	0.675		
Responding parents’ employment status	Employed	9 (3.6%)	117 (46.4%)	126 (50.0%)	–	–	0.065	0.596‡
	Unemployed	0 (0.0%)	2 (50.0%)	2 (50.0%)	NE	–		
	Househusband	0 (0.0%)	7 (33.3%)	14 (66.7%)	2.06 (0.95–4.44)	0.065		
Spouses/cohabitants’ employment status	Employed	9 (3.5%)	114 (43.8%)	137 (52.7%)	–	–	0.074	0.122‡
	Unemployed	0 (0.0%)	11 (78.6%)	3 (21.4%)	0.35 (0.11–1.11)	0.074		
	Househusband	0 (0.0%)	1 (33.3%)	2 (66.7%)	NE	–		
Responding parents’ citizenship	Italian	9 (3.4%)	122 (45.5%)	137 (51.1%)	NE	–		1.000‡
	Not Italian	0 (0.0%)	4 (44.4%)	5 (55.6%)	NE			
Responding parents’ religious orientation	Catholic Christian	6 (2.6%)	107 (45.7%)	121 (51.7%)	–	–	0.587	0.348‡
	Orthodox Christian	0 (0.0%)	1 (50.0%)	1 (50.0%)	NE	–		
	Atheist	3 (12.0%)	11 (44.0%)	11 (44.0%)	0.61 (0.23–1.61)	0.306		
	Other religions/not responding	0 (0.0%)	7 (43.8%)	9 (56.2%)	1.24 (0.49–3.16)	0.642		
Responding parents’ political orientation (Optional) (147)	Left-wing political orientation	4 (7.3%)	28 (50.9%)	23 (41.8%)	–	–	0.204	0.460‡
	Centrist political orientation	3 (5.3%)	27 (47.4%)	27 (47.4%)	1.28 (0.53–3.09)	0.573		
	Right-wing political orientation	0 (0.0%)	15 (42.9%)	20 (57.1%)	2.02 (0.92–4.47)	0.079		
Parental health literacy–IMETER	Low literacy	2 (3.2%)	33 (52.4%)	28 (44.4%)	–	–	0.493	0.664‡
	Marginal literacy (limited)	5 (3.0%)	70 (42.4%)	90 (54.5%)	1.46 (0.76–2.83)	0.251		
	Functional literacy (adequate)	2 (4.1%)	23 (46.9%)	24 (49.0%)	1.16 (0.60–2.25)	0.651		

The ordinal OR estimates the odds of being in a higher judgment category. The first eligible predictor category is the reference. OR confidence intervals, OR *p*-values and the omnibus Wald *p*-value use HC1 robust standard errors clustered by school (R), with t/F reference distributions based on G − 1 degrees of freedom. The school-clustered overall *p*-value is the primary omnibus Wald F test. The secondary categorical *p*-value uses Pearson’s chi-square test when every expected count is ≥5, Fisher’s exact test for sparse 2 × 2 tables (†), or Fisher’s test with Monte Carlo simulation for sparse R × C tables (‡; B = 100,000). The secondary test ignores outcome ordering and school clustering. Categories with fewer than 10 complete observations are shown descriptively but excluded from ordinal regression; their OR is reported as NE. NE, not estimable. CI, confidence interval; OR, odds ratio.

**Table 7:** Scale D (Health-related knowledge).

Characteristic	Level	Low	Moderate	High	Ordinal OR (95% CI)	OR *p*-value	School-clustered overall *p*-value	Secondary categorical *p*-value
Adolescents								
Sex (293)	Female	81 (52.6%)	63 (40.9%)	10 (6.5%)	–	–	0.217	0.421
	Male	65 (46.8%)	60 (43.2%)	14 (10.1%)	1.31 (0.85–2.01)	0.217		
Province (340)	Terni	24 (57.1%)	15 (35.7%)	3 (7.1%)	–	–	0.548	0.715‡
	Perugia	151 (50.7%)	124 (41.6%)	23 (7.7%)	1.27 (0.57–2.85)	0.548		
Responding parents (277)								
Parentage	Mother	129 (50.8%)	105 (41.3%)	20 (7.9%)	–	–	0.306	0.448‡
	Father	9 (39.1%)	11 (47.8%)	3 (13.0%)	1.64 (0.63–4.29)	0.306		
Responding parents’ educational attainment	Up to high school	84 (52.8%)	62 (39.0%)	13 (8.2%)	–	–	0.416	0.490
	University degree or over	54 (45.8%)	54 (45.8%)	10 (8.5%)	1.28 (0.70–2.35)	0.416		
Spouses/cohabitants’ educational attainment	Up to high school	111 (50.7%)	94 (42.9%)	14 (6.4%)	–	–	0.331	0.099‡
	University degree or over	27 (46.6%)	22 (37.9%)	9 (15.5%)	1.38 (0.71–2.69)	0.331		
Responding parents’ employment status	Employed	125 (49.6%)	105 (41.7%)	22 (8.7%)	–	–	0.381	0.751‡
	Unemployed	1 (25.0%)	3 (75.0%)	0 (0.0%)	NE	–		
	Househusband	12 (57.1%)	8 (38.1%)	1 (4.8%)	0.72 (0.33–1.54)	0.381		
Spouses/cohabitants’ employment status	Employed	129 (49.6%)	110 (42.3%)	21 (8.1%)	–	–	0.951	0.401‡
	Unemployed	7 (50.0%)	6 (42.9%)	1 (7.1%)	0.97 (0.36–2.64)	0.951		0.678‡
	Househusband	2 (66.7%)	0 (0.0%)	1 (33.3%)	NE	–		
Responding parents’ citizenship	Italian	133 (49.6%)	113 (42.2%)	22 (8.2%)	NE	–		
	Not Italian	5 (55.6%)	3 (33.3%)	1 (11.1%)	NE	–		
Responding parents’ religious orientation	Catholic Christian	116 (49.6%)	101 (43.2%)	17 (7.3%)	–	–	0.833	0.455‡
	Orthodox Christian	2 (100.0%)	0 (0.0%)	0 (0.0%)	NE	–		
	Atheist	13 (52.0%)	8 (32.0%)	4 (16.0%)	1.09 (0.38–3.12)	0.869		
	Other religions/not responding	7 (43.8%)	7 (43.8%)	2 (12.5%)	1.35 (0.48–3.82)	0.563		
Responding parents’ political orientation (Optional) (147)	Left-wing political orientation	30 (54.5%)	21 (38.2%)	4 (7.3%)	–	–	0.414	0.069‡
	Centrist political orientation	25 (43.9%)	23 (40.4%)	9 (15.8%)	1.72 (0.75–3.94)	0.191		
	Right-wing political orientation	16 (45.7%)	19 (54.3%)	0 (0.0%)	1.19 (0.64–2.19)	0.575		
Parental health literacy–IMETER	Low literacy	40 (63.5%)	21 (33.3%)	2 (3.2%)	–	–	0.017	0.017‡
	Marginal literacy (limited)	81 (49.1%)	71 (43.0%)	13 (7.9%)	1.84 (1.07–3.16)	0.029		
	Functional literacy (adequate)	17 (34.7%)	24 (49.0%)	8 (16.3%)	3.57 (1.49–8.56)	0.005		

The ordinal OR estimates the odds of being in a higher judgment category. The first eligible predictor category is the reference. OR confidence intervals, OR *p*-values and the omnibus Wald *p*-value use HC1 robust standard errors clustered by school (R), with t/F reference distributions based on G − 1 degrees of freedom. The school-clustered overall *p*-value is the primary omnibus Wald F test. The secondary categorical *p*-value uses Pearson’s chi-square test when every expected count is ≥5, Fisher’s exact test for sparse 2 × 2 tables (†), or Fisher’s test with Monte Carlo simulation for sparse R × C tables (‡; B = 100,000). The secondary test ignores outcome ordering and school clustering. Categories with fewer than 10 complete observations are shown descriptively but excluded from ordinal regression; their OR is reported as NE. NE, not estimable. CI, confidence interval; OR, odds ratio.

## 4 Discussion

The present study evaluated four domains of health literacy among early adolescents in the Umbria Region and explored their associations with selected socio-demographic and cultural characteristics. Across the four domains assessed by the MOHLAA-Q, the observed responses indicated a high proportion of reported difficulties in dealing with health-related information and a high proportion of participants classified in the low category for health-related knowledge. Previous research has highlighted adolescence as an important period for the development of health-related knowledge, skills, and decision-making capacities (11), while studies among Italian students have also highlighted the importance of monitoring health literacy and its social and school-related determinants (17). This literature provides relevant context for interpreting the present findings and identifying areas that may warrant further investigation. Although the generalizability of our findings is limited, the concerns observed in specific domains of adolescents’ health literacy may help inform further research and potentially targeted interventions. The exploratory analyses suggested that the socio-demographic and cultural characteristics considered in this study were not uniformly associated with the different dimensions of adolescents’ health literacy. Rather, the observed pattern varied across domains, supporting the view that health literacy is a multidimensional construct and that its different components may be shaped by different individual and family-level factors (14). For the domain concerning the management of health-related information (Scale A), an association with adolescent sex was observed, with males showing a greater likelihood of reporting fewer difficulties than females. This finding differs from the direction reported in some previous studies. In particular, the German study in which the MOHLAA-Q was developed and applied to adolescents aged 14–17 years reported higher health-literacy levels among females (9). However, comparisons across studies should be interpreted cautiously because of differences in age, population characteristics, cultural context, health-literacy measures, and outcome definitions. More broadly, evidence on adolescent health literacy does not support a uniform pattern of sex differences across populations and health-literacy dimensions (10). The present finding may therefore reflect characteristics specific to the early-adolescent developmental stage or to the particular domain assessed, rather than a general difference in health literacy between males and females. In addition, because the present analyses were exploratory and univariable, the observed association may reflect other characteristics correlated with sex that were not measured or controlled for. Further studies using age-appropriate instruments and comparable multidimensional measures are needed to determine whether sex-related differences in the ability to deal with health-related information are consistent across developmental stages and settings. The same domain also showed a relationship with the educational attainment of the responding parent’s spouse/cohabitant. Rather than suggesting a direct effect of parental education, this finding may reflect the broader influence of the family environment on adolescents’ opportunities to access, discuss, and make sense of health-related information. Educational resources within the family may contribute to the availability of health-related information and to patterns of communication and support (14), although the mechanisms underlying this association remain unclear. Importantly, the absence of comparable associations across the other domains suggests that family educational characteristics may be more closely related to some aspects of health literacy than to others. For communication and interaction skills (Scale B), the observed association with the employment status of the responding parent should be interpreted cautiously. The finding does not readily support a specific explanatory pathway and may reflect characteristics of the family context that were not measured in the present study. Moreover, the small number of participants in some employment categories limits the interpretability and stability of this result. It is therefore more appropriately considered a hypothesis-generating finding rather than evidence of a meaningful relationship between parental employment and adolescents’ communication skills. No clear socio-demographic or cultural pattern emerged for attitudes toward one’s own health and health information (Scale C). Similarly, the absence of consistent associations across several characteristics and domains suggests that the factors examined in this study do not provide a simple explanation for variation in adolescents’ health literacy. This may reflect the multidimensional nature of the construct but may also be related to the characteristics and size of the study sample and to the measurement limitations discussed below. An interesting pattern emerged for the health-related knowledge score (Scale D), for which higher levels of parental health literacy were associated with higher adolescent knowledge-score categories. This finding is in line with previous research suggesting a relationship between parental and adolescent health literacy (23). The family may represent an important context in which health-related knowledge is acquired, discussed, and reinforced, and parents with greater health literacy may be better positioned to support adolescents in accessing and interpreting health information. However, the present findings cannot establish that parental health literacy directly determines adolescents’ knowledge. Shared educational or socio-economic circumstances, family communication patterns, or other unmeasured characteristics could contribute to the observed association. Longitudinal studies would therefore be valuable to clarify the direction and mechanisms of this relationship. The additional psychometric analyses conducted in the present sample showed substantial variability in the performance of the four MOHLAA-Q scales. Scales A and B showed comparatively higher internal consistency and generally positive item-total correlations, whereas Scales C and D showed lower internal consistency and weaker item-level performance. The confirmatory factor analysis also provided mixed evidence regarding the intended correlated four-factor structure, with acceptable RMSEA but CFI, TLI, and SRMR values that did not uniformly meet the predefined approximate criteria. These findings provide important context for interpreting the domain-specific results. The findings for Scale D were particularly relevant. The low KR-20 coefficient, limited item discrimination, and generally weak standardized factor loadings provide limited evidence of internal coherence and of a homogeneous latent structure for the dichotomous knowledge items in this sample. However, Scale D was originally designed as a health-related knowledge score based on the number of correct answers to questions covering different predefined content areas, rather than as a reflective scale in which all items are expected to be interchangeable indicators of a single latent construct. Therefore, the low internal consistency does not by itself invalidate the use of the predefined knowledge score, but it limits the extent to which Scale D can be interpreted as a homogeneous latent health-knowledge construct. Accordingly, the findings involving Scale D, including the observed associations with parental health literacy, should be interpreted as exploratory associations with categories of the health-related knowledge score and not as evidence of associations with a validated unidimensional latent trait. The study findings should be interpreted considering several limitations. First, the cross-sectional design did not allow any conclusion to be drawn regarding the temporal or causal role of socio-demographic and cultural variables. Several aspects related to the recruitment process and the study population as well as to the tools used to collect information should be taken into account. The two-stage voluntary recruitment process may have introduced selection bias. At the school level, participating schools may have differed systematically from non-participating schools, while at the individual level adolescents and responding parents who completed the questionnaire may have differed from those who did not participate. Because sufficiently detailed administrative or contextual information on non-participating schools was not available, comparisons according to participating and non-participating schools could not be performed. Similarly, comparable individual-level characteristics of responding and non-responding adolescents and responding parents were not available. Therefore, the direction and magnitude of potential selection bias could not be quantified. Accordingly, the findings should be interpreted with caution in terms of generalizability to the wider population of adolescents in Umbria because the study sample could be not representative of all adolescents. This potential lack of generalizability could be also due to potential selection bias related to the use of the electronic school register as recruitment channel. Although the electronic school register is routinely used for school–family communication in Italy, its use as a recruitment channel may have preferentially facilitated participation among families with reliable digital access, greater familiarity with electronic communication, higher digital literacy, or greater engagement with school communications. This issue is particularly relevant because these characteristics may themselves be associated with the ability to access, understand, evaluate, and use health-related information (7). Moreover, the online and self-administered mode of questionnaire completion may have introduced variability in respondents’ engagement with the questions and in the way response options were interpreted, which may have contributed to measurement variability. The findings should consequently be interpreted cautiously with regard to their generalizability to families with lower digital access or digital literacy. A further source of potential selection bias relates to the requirement that at least one parent be proficient in Italian. Although this criterion was adopted to ensure adequate comprehension of the questionnaire and informed consent procedures, it may have resulted in the underrepresentation of some families limiting the generalizability of the findings to linguistically and culturally diverse families. Because information on families who did not participate was not available, the extent of this potential bias cannot be determined. Eventually, generalizability is limited by the low response rate and the skewed distribution of the sample in terms of province of the attended school. An additional contextual dimension that could not be examined in the present study is the distinction between rural and urban areas. The province of the attended school was recorded, but a specific measure of urbanicity or rurality was not collected; therefore, the two provinces cannot be interpreted as proxies for urban and rural populations. This distinction may nevertheless be relevant when interpreting health-literacy findings, as previous research has reported differences between rural and urban populations, although the evidence suggests that such differences may be partly explained by education, socioeconomic conditions, access to services, and other sociodemographic characteristics rather than by rurality itself (24). Consequently, the present study cannot determine whether the observed patterns differ according to the degree of urbanization or rurality within Umbria. Future studies should explicitly collect information on residential context and examine rural–urban differences alongside socioeconomic, educational, and access-related factors. With respect to the measurement instrument, several limitations should also be considered. The MOHLAA-Q was originally developed and validated in an older adolescent population (14–17 years) than that included in the present study. As individual chronological age was not collected, eligibility was defined according to school grade, and the age distribution of the participants cannot be established. Therefore, differences in item comprehension, response processes, and the functioning of response thresholds cannot be excluded, and the psychometric properties established in the original validation study cannot be assumed to be fully transferable to the present population. These considerations are also relevant to the use of ordinal response scales, as differences in the interpretation of response categories may affect how adolescents, particularly those in a younger age group, position themselves across adjacent categories. Consequently, the observed category distributions may partly reflect differences in response processes in addition to differences in the underlying health-literacy domains. The applicability of the questionnaire thresholds to this younger population should therefore also be interpreted with caution. Furthermore, the Italian version of the MOHLAA-Q used in this study was translated by the research team, without formal back-translation or cognitive interviewing, which may have affected linguistic and cultural equivalence and contributed to differences in item functioning. Accordingly, the additional psychometric analyses conducted in the present sample should be considered sample-specific and exploratory and were not intended to constitute a formal validation of the Italian version of the MOHLAA-Q. The findings obtained using the MOHLAA-Q should therefore be interpreted cautiously, including with regard to potential misclassification arising from the use of a self-report questionnaire and from the applicability of the instrument and its thresholds to this younger population. With respect to the statistical analyses, some contingency-table cells were sparse, which may have reduced the precision and statistical power of the analyses. In addition, the number of statistical comparisons increases the possibility of chance findings. The ordinal regression analyses were conducted separately for each predictor and therefore did not provide estimates adjusted simultaneously for potential confounding factors. Consequently, the observed associations should not be interpreted as independent or causal effects. The exploratory nature of the analyses, together with the sample size and sparse categories, means that the identified associations require confirmation in larger and more representative samples and in studies incorporating multivariable approaches and designs that allow potential confounding and temporal relationships to be more appropriately assessed. Notwithstanding these limitations, the study contributes to addressing a gap in knowledge on adolescents’ health literacy in the Italian context by providing descriptive evidence on specific dimensions of health literacy and identifying exploratory associations between selected socio-demographic and cultural characteristics and specific health-literacy domains (17, 18). The findings may help identify specific components of health literacy that could be considered in future research and health-promotion initiatives. They also suggest possible differences across specific health-literacy domains according to adolescent sex, family educational context, parental employment status, and parental health literacy. However, these exploratory associations require confirmation in larger and more representative studies using age-appropriate and adequately validated instruments.

## Data Availability

The datasets generated and analysed during the current study are not publicly available because they include individual-level information collected from minors and their parents and are subject to applicable ethical and data-protection requirements. De-identified individual-level data may be made available from the corresponding author on reasonable request, subject to appropriate ethical and data-protection requirements and the conditions of the ethical approval under which the study was conducted. Aggregated data are reported in the manuscript and supplementary materials. The complete adolescent and parent questionnaires, including response options and scoring procedures, are provided as supplementary material.
