# Supplementary material for: Health literacy among early adolescents in Umbria, Italy: a cross-sectional study
Source: Front Public Health. 2026 Oct 27;14:1925432. doi: 10.3389/fpubh.2026.1925432 (PMC13748303; doi:10.3389/fpubh.2026.1925432)
Supplement: Supplementary file 1 [file Data_Sheet_1.PDF]

Presentazione dello Studio

Gentile Genitore, chiediamo la Sua disponibilità e quella di Suo/a Figlio/a a partecipare ad un progetto di ricerca, condotto dalla Prof.ssa Chiara de Waure, Professoressa Associata del Dipartimento di Medicina e Chirurgia dell'Università degli Studi di Perugia.

L'obiettivo di questo studio è duplice:

1. **Determinare quanti adolescenti hanno fatto la vaccinazione contro l'HPV rispetto agli altri vaccini raccomandati per età;**
2. **Studiare il ruolo di alcuni determinanti individuali, socio-economici e culturali nell'uptake del vaccino HPV e degli altri vaccini raccomandati per età.**

I risultati del progetto potranno fornire nuove conoscenze su determinanti ad oggi meno studiati contribuendo a progettare interventi su misura per aumentare la copertura vaccinale, in particolar modo per HPV, anche alla luce dell'obiettivo stabilito dall'Organizzazione Mondiale della Sanità per il raggiungimento dell'eliminazione del tumore della cervice uterina.

La invitiamo pertanto a compilare, insieme a Suo/a Figlio/a, il questionario che troverà di seguito. La prima parte, dalla Domanda 6 alla Domanda 23, richiede la compilazione da parte di Suo/a Figlio/a, mentre per la sezione del consenso e del trattamento dei dati, e dalla Domanda 24 in poi, chiediamo il Suo contributo.

È importante che valutiate tutte le affermazioni. Non ci sono risposte giuste o sbagliate. La compilazione del

questionario richiederà circa 20 minuti del Vostro tempo.

La Vostra partecipazione è assolutamente volontaria, siete liberi di non compilare il questionario o di non inviarlo senza alcuna penalità o ripercussione. Tuttavia Vi saremo molto grati se voleste collaborare inviando il questionario compilato in ogni sua parte. Noi Vi garantiamo la tutela dell'anonimato e la confidenzialità secondo le Norme vigenti.

Cliccando su questo link potete trovare una breve presentazione del Progetto.

Il Protocollo di Studio è stato redatto secondo una rigida metodologia scientifica ed è stato sottoposto al Comitato Etico Regionale dell'Umbria che ha dato Parere favorevole allo svolgimento.

Per informazioni o eventuali chiarimenti di carattere scientifico è possibile contattare il Responsabile scientifico della ricerca al seguente riferimento E-mail: chiara.dewaure@unipg.it

Per informazioni o eventuali segnalazioni/richieste di chiarimento tecniche sul questionario scrivere a vito.cerabona@unipg.it

Cordialmente Prof.ssa Chiara de Waure

Consenso informato e Trattamento dei dati - Compilazione a cura del **Genitore**

Gentile Genitore, di seguito la sezione inerente

all'acquisizione del consenso informato e le informative redatte ai sensi del D. Lgs. n. 196 del 30/06/2003 e Linee Guida per i trattamenti dei dati personali nell'ambito degli Studi alla luce del nuovo regolamento generale sulla protezione dei dati (General Data Protection Regulation GDPR).

* 1. Tipo di parentela/relazione con lo/a studente/ssa

* 2. Per quanto concerne i termini contenuti nel Documento Trattamento Dati Genitori (Clicca sul link)

Nel caso non si dovesse aprire il file cliccare su 'Aggiorna', qualora la problematica dovesse persistere è possibile scrivere a **vito.cerabona@unipg.it** ed una copia del Documento trattamento dati Le verrà inviata via mail.

- Do il Consenso
- Non do il Consenso

* 3. Per quanto concerne i termini contenuti nel Documento Trattamento dati Figlio (Clicca sul link)

Nel caso non si dovesse aprire il file cliccare su 'Aggiorna', qualora la problematica dovesse persistere è possibile scrivere a **vito.cerabona@unipg.it** ed una copia del Documento trattamento dati Le verrà inviata via mail.

- Do il mio Consenso
- Non do il mio Consenso

* 4. Per quanto concerne i termini contenuti nel Documento Foglio informativo per il partecipante (Clicca sul link)

Nel caso non si dovesse aprire il file cliccare su 'Aggiorna', qualora la problematica dovesse persistere è possibile scrivere a **vito.cerabona@unipg.it** ed una copia del Documento Foglio informativo per il partecipante Le verrà inviata via mail.

- Do il mio Consenso
- Non do il mio Consenso

Per rispondere alle domande è obbligatorio dare il consenso in tutti i campi. La non accettazione di tutti i termini sopra proposti porterà alla fine del questionario.

Prima parte - Compilazione a cura dello/a **Studente/ssa**

Gentile Studente/ssa di seguito troverai un questionario che ti preghiamo di compilare in autonomia fino alla Domanda 23. Ti ringraziamo per la disponibilità.

Prima di iniziare, cliccando su questo link puoi trovare una scheda per l'Assenso al prosieguo del questionario.

Nel caso non si dovesse aprire il file cliccare su 'Aggiorna', qualora la problematica dovesse persistere è possibile scrivere a vito.cerabona@unipg.it ed una copia del Documento Assenso per Adolescente ti verrà inviata via mail.

* 5. Assenso a proseguire il questionario

- ○ Do il mio assenso
- ○ Non do il mio assenso

Istituto umbro d'appartenenza - Compilazione a cura dello/a **Studente/ssa**

* 6. Indica il tuo Istituto d'appartenenza

7. Quale è il tuo Genere?

- ○ Femmina
- ○ Maschio
- ○ Preferisco non rispondere

Sezione alfabetizzazione sanitaria - Compilazione a cura dello/a **Studente/ssa**

* 8. Quanto è facile/difficile per te?

	Molto Facile	Facile	Difficile	Molto Difficile
Trovare informazioni su cosa fare per stare meglio quando ti senti male	○	○	○	○
Comprendere il foglietto illustrativo di un farmaco	○	○	○	○
Valutare se le informazioni sui farmaci nei media sono credibili (media: internet, TV, radio, stampa)	○	○	○	○
Seguire le istruzioni del tuo medico o farmacista	○	○	○	○
Trovare informazioni su come affrontare problemi psicologici (Ad esempio, stress permanente, depressione, essere vittima di bullismo, disturbi alimentari)	○	○	○	○
Comprendere come proteggerti dalle malattie sessualmente trasmissibili (Per malattie sessualmente trasmissibili intendiamo malattie come l'HIV/AIDS, l'infezione da clamidia o l'herpes)	○	○	○	○
Valutare se puoi fidarti dei media quando ti avvertono dei rischi per la tua salute	○	○	○	○
Mettere in pratica le raccomandazioni che ti proteggono durante lo sport, nel tempo libero e nel traffico (Ad esempio, indossare un casco o protezioni per ginocchia o braccia, osservare le regole del codice stradale)	○	○	○	○

	Molto Facile	Facile	Difficile	Molto Difficile
Trovare informazioni su comportamenti salutari come l'esercizio fisico e l'alimentazione	◯	◯	◯	◯
Comprendere le informazioni sulla confezione dei prodotti alimentari	◯	◯	◯	◯
Valutare come ciò che fai quotidianamente influisce sulla tua salute (Ad esempio, alimentazione, consumo di liquidi, esercizio fisico, relax, cura del corpo)	◯	◯	◯	◯
Coinvolgerti nella promozione di un ambiente salutare (Ad esempio, meno rumore e meno traffico, migliore qualità dell'aria, più parchi e campi sportivi)	◯	◯	◯	◯

* 9. In che misura sei d'accordo con le seguenti frasi?

	Totalmente d'accordo	Abbastanza d'accordo	Abbastanza in disaccordo	Totalmente in disaccordo
Durante la mia ultima visita dal medico, ho fatto tutte le domande che mi interessavano	◯	◯	◯	◯
Chiacchiero con i miei amici su come evitare comportamenti non salutari, come fumare o bere alcolici in eccesso	◯	◯	◯	◯
Se i miei amici o i miei fratelli hanno domande sulla salute, posso aiutarli	◯	◯	◯	◯
È facile per me parlare con i miei genitori di argomenti legati alla salute	◯	◯	◯	◯
In generale presto molta attenzione alla mia salute	◯	◯	◯	◯

* 10. In che misura sei d'accordo con le seguenti frasi?

	Totalmente d'accordo	Abbastanza d'accordo	Né d'accordo né in disaccordo,	Abbastanza in disaccordo	Totalmente in disaccordo
Chiedo consigli agli altri quando sono malato	○	○	○	○	○
Sta a me proteggermi dalle malattie	○	○	○	○	○
So cosa devo fare per sentirmi a mio agio	○	○	○	○	○
È importante per me informarmi su argomenti legati alla salute	○	○	○	○	○

* 11. Quanto spesso un giovane della tua età dovrebbe essere fisicamente attivo?

- ○ Due ore di sport alla settimana sono sufficienti
- ○ Le lezioni di educazione fisica a scuola sono sufficienti
- ○ Circa un'ora al giorno, sudando, è sufficiente
- ○ Fare esercizio tre volte alla settimana è sufficiente
- ○ Non lo so

* 12. Come influisce sul corpo il consumo regolare di grandi quantità di alcol?

- ○ Vengono danneggiati quasi tutti gli organi
- ○ Vengono danneggiati solo il fegato e il cervello
- ○ Dipende da quanto il corpo può tollerare
- ○ Diminuisce il volume dei polmoni
- ○ Non lo so

* 13. Quali sono gli effetti sulla salute per i giovani che consumano spesso cannabis (marijuana, hashish)?

- ○ Peggioramento delle capacità di apprendimento e memoria
- ○ Danneggiamento permanente del cervello
- ○ Perdita dei capelli
- ○ Solo dipendenza fisica
- ○ Non lo so

* 14. Quale NON è uno dei possibili effetti del fumo?

- ○ Perdita dei denti
- ○ Invecchiamento della pelle
- ○ Miopia
- ○ Diminuzione della forza muscolare
- ○ Non lo so

* 15. Come possono essere trattate le piccole ustioni?

- ◯ Cospargere la ferita di farina
- ◯ Raffreddare per 10 minuti con acqua tiepida
- ◯ Sfiorare sempre le vesciche delle ustioni
- ◯ Raffreddare solo con soluzione salina sterile
- ◯ Non lo so

* 16. Quale ingrediente è contenuto in maggior quantità in una bevanda al cacao in polvere con i seguenti ingredienti elencati sulla confezione: zucchero, destrosio, cacao in polvere a basso contenuto di grassi, agente emulsionante (lecitina), sale?

- ◯ Sale
- ◯ Destrosio
- ◯ Zucchero
- ◯ Cacao in polvere
- ◯ Non lo so

* 17. Vogliamo sapere se conosci i tuoi diritti. Una delle seguenti frasi è SBAGLIATA. Quale?

- ◯ Posso oppormi a un'importante operazione chirurgica, anche se i miei genitori hanno acconsentito
- ◯ Devo acconsentire affinché il mio medico informi i miei genitori sul mio trattamento
- ◯ Ho il diritto che il medico mi informi sui vantaggi e sugli svantaggi del mio trattamento
- ◯ Non ho diritti
- ◯ Non lo so

* 18. Come può essere trasmesso l'HIV/AIDS?

- ◯ Tramite il bacio
- ◯ Tramite il contatto con la pelle (es. stringendo la mano)
- ◯ Con le punture di insetti
- ◯ Nessuna delle risposte precedenti è corretta
- ◯ Non lo so

Conoscenze sul Virus HPV - Compilazione a cura dello/a **Studente/ssa**

Il seguente questionario andrà a valutare le tue conoscenze riguardo al Papilloma Virus Umano (HPV) e alle malattie a esso correlate.

* 19. **Conoscenze**

Per favore indica quanto sei d’accordo con ciascuna affermazione.

	Fortemente in disaccordo	In disaccordo	Né d'accordo né in disaccordo	D'accordo	Fortemente d'accordo
I maschi non possono contrarre l’HPV	◯	◯	◯	◯	◯

	Fortemente in disaccordo	In disaccordo	Né d'accordo né in disaccordo	D'accordo	Fortemente d'accordo
Le donne vaccinate contro HPV devono sottoporsi a pap test (controlli per il tumore cervicale) con il crescere dell'età	○	○	○	○	○
Si può contrarre l'HPV tramite attività sessuale	○	○	○	○	○
L'HPV è molto raro	○	○	○	○	○
L'HPV può provocare un tumore cervicale	○	○	○	○	○
Il vaccino per HPV protegge contro tutti i tipi di tumore cervicale	○	○	○	○	○

Opinioni nei confronti dei Vaccini - Compilazione a cura dello/a **Studente/ssa**

Il seguente questionario va ad analizzare i pensieri generali che le persone possono avere nei confronti dei vaccini, vagliando diversi aspetti.

Per favore seleziona un elemento nella scala a 6 punti (da fortemente in disaccordo a fortemente d'accordo).

* 20. Sfiducia nel vaccino

	Fortemente in disaccordo					Fortemente d'accordo
Mi sento sicuro/a dopo essere stato vaccinato/a	○	○	○	○	○	○
Confido nei vaccini per debellare gravi malattie infettive	○	○	○	○	○	○
Mi sento protetto/a dopo essere stato/a vaccinato/a	○	○	○	○	○	○

* 21. Preoccupazione riguardo effetti imprevisti futuri

	Fortemente in disaccordo					Fortemente d'accordo
Sebbene molti vaccini sembrino sicuri, ci potrebbero essere complicanze non ancora note	○	○	○	○	○	○
I vaccini possono causare problemi imprevisti nei bambini	○	○	○	○	○	○

	Fortemente in disaccordo					Fortemente d'accordo
Mi preoccupo per futuri effetti sconosciuti dei vaccini	◯	◯	◯	◯	◯	◯

* 22. Riserve riguardo il profitto commerciale

	Fortemente in disaccordo					Fortemente D'accordo
I vaccini producono molto fatturato per le aziende farmaceutiche ma pochi benefici alle persone	◯	◯	◯	◯	◯	◯
Le Autorità promuovono la vaccinazione per profitto, non per la salute delle persone	◯	◯	◯	◯	◯	◯
I programmi di vaccinazione sono un grande complotto	◯	◯	◯	◯	◯	◯

* 23. Preferenza per l'immunità naturale (acquisita successivamente a contrazione di malattia)

	Fortemente in disaccordo					Fortemente d'accordo
L'immunità naturale dura più a lungo di una vaccinazione	◯	◯	◯	◯	◯	◯
L'esposizione naturale a virus e batteri offre la protezione più sicura	◯	◯	◯	◯	◯	◯
Essere naturalmente esposti alle malattie è più sicuro per il sistema immunitario che essere esposti attraverso la vaccinazione	◯	◯	◯	◯	◯	◯

Seconda parte - Compilazione da parte dei **Genitori**

Gentile Genitore, da questo punto in poi La invitiamo a rispondere alle domande del questionario che troverà di seguito.

Sezione socio-anagrafica - Compilazione da parte dei **Genitori**

* 24. Il questionario è compilato da

◯ Padre

◯ Madre

* 25. Qual è il Suo titolo di studio più elevato?

- ◯ Nessuno
- ◯ Scuola elementare
- ◯ Scuola media
- ◯ Scuola superiore
- ◯ Laurea
- ◯ Post-laurea

* 26. Qual è il titolo di studio più elevato di Suo/a marito/moglie/convivente?

- ◯ Nessuno
- ◯ Scuola elementare
- ◯ Scuola media
- ◯ Scuola superiore
- ◯ Laurea
- ◯ Post-laurea
- ◯ Non applicabile

* 27. Qual è la Sua attività lavorativa?

- ◯ Dipendente full-time
- ◯ Dipendente part-time
- ◯ Libero professionista/Lavoratore Autonomo
- ◯ Casalingo/a
- ◯ In cerca di occupazione
- ◯ Disoccupato
- ◯ Altro (specificare)

* 28. Qual è l'attività lavorativa di Suo/a marito/moglie/convivente?

- ◯ Dipendente full-time
- ◯ Dipendente part-time
- ◯ Libero professionista/Lavoratore Autonomo
- ◯ Casalingo/a
- ◯ In cerca di occupazione
- ◯ Disoccupato
- ◯ Non applicabile

* 29. Per favore indichi la sua cittadinanza

- Italiana
- Altro (specificare)

* 30. Per favore indichi a quale etnia (per continente) appartiene?

- America settentrionale
- America Meridionale
- Europa
- Africa
- Asia
- Oceania
- Preferisco non rispondere
- Altro (specificare)

* 31. Per favore indichi la sua confessione religiosa

- Cristiana cattolica
- Cristiana ortodossa
- Musulmana
- Ebraica
- Cristiana protestante
- Buddhista
- Induista
- Preferisco non rispondere
- Altro (specificare)

* 32. Suo/a figlio/a ha effettuato il richiamo vaccinale per Tetano-Difterite-Pertosse-Polio in età adolescenziale?

- Sì
- No
- Non ricordo

* 33. Suo/a figlio/a ha effettuato il richiamo vaccinale per Meningococco tetravalente in età adolescenziale?

- Sì
- No
- Non ricordo

* 34. Suo/a figlio/a ha effettuato il vaccino per il Papilloma Virus (HPV) in età adolescenziale?

- ◯ Si, una dose
- ◯ Si, due dosi
- ◯ No
- ◯ Non ricordo

35. In politica, molte persone usano i termini "sinistra" e "destra". Su una scala da 0 (fortemente di sinistra) a 10 (fortemente di destra) dove colloca le Sue convinzioni politiche? La domanda non è obbligatoria e può decidere di non rispondere.

0	1	2	3	4	5	6	7	8	9	10
◯	◯	◯	◯	◯	◯	◯	◯	◯	◯	◯

Influenza culturale - Compilazione da parte dei **Genitori**

In questa parte del questionario, Le verrà chiesto come percepisce che il Suo contesto culturale (appartenenza culturale) influenzi vari aspetti della Sua vita e delle persone a Lei vicine.

* 36. In che misura l'appartenenza culturale influisce sui seguenti elementi?

	Molto poco/mai	Poco/ogni tanto	Moderatamente/qualche volta	Tanto/spesso	Tantissimo
i vestiti che indossa	◯	◯	◯	◯	◯
lo stile dei Suoi capelli	◯	◯	◯	◯	◯
i gioielli che preferisce	◯	◯	◯	◯	◯
le decorazioni della Sua casa	◯	◯	◯	◯	◯
le Sue scelte di cibo e bevande	◯	◯	◯	◯	◯
come festeggia le festività in generale	◯	◯	◯	◯	◯
se e come festeggia le festività specifiche della Sua cultura	◯	◯	◯	◯	◯
la lingua (o le lingue) che parla a casa	◯	◯	◯	◯	◯
la lingua (o le lingue) che parla al di fuori di casa	◯	◯	◯	◯	◯
la TV e la musica che preferisce	◯	◯	◯	◯	◯

	Molto poco/mai	Poco/ogni tanto	Moderatamente/qualche volta	Tanto/spesso	Tantissimo
come gli uomini e le donne si relazionano tipicamente tra loro nel Suo gruppo culturale (uguale o diverso)	○	○	○	○	○
come si attiene al modo di comportarsi del Suo gruppo culturale nelle relazioni uomo/donna	○	○	○	○	○
come i genitori nel Suo gruppo culturale tipicamente si relazionano con i loro figli	○	○	○	○	○
come si attiene al modo di comportarsi del Suo gruppo culturale nelle relazioni genitore/figlio	○	○	○	○	○
quante persone nella Sua zona condividono la Sua appartenenza culturale	○	○	○	○	○
quanti dei Suoi amici condividono la Sua appartenenza culturale	○	○	○	○	○
con quale frequenza incontra un guaritore popolare del Suo gruppo culturale	○	○	○	○	○
con quale frequenza usa erbe o medicine speciali comuni nel Suo gruppo culturale	○	○	○	○	○

Opinioni nei confronti dei Vaccini - Compilazione da parte dei **Genitori**

Il seguente questionario va ad analizzare i pensieri generali che le persone possono avere nei confronti dei vaccini, vagliando diversi aspetti.

Per favore selezioni un elemento sulla scala a 6 punti (da fortemente in disaccordo a fortemente d'accordo).

* 37. Sfiducia nel vaccino

	Fortemente in disaccordo					Fortemente d'accordo
Mi sento sicuro/a dopo essere stato vaccinato/a	○	○	○	○	○	○
Confido nei vaccini per debellare gravi malattie infettive	○	○	○	○	○	○
Mi sento protetto/a dopo essere stato/a vaccinato/a	○	○	○	○	○	○

* 38. Preoccupazione riguardo effetti imprevisti futuri

	Fortemente in disaccordo					Fortemente d'accordo
Sebbene molti vaccini sembrino sicuri, ci potrebbero essere complicanze non ancora note	○	○	○	○	○	○
I vaccini possono causare problemi imprevisti nei bambini	○	○	○	○	○	○
Mi preoccupo per futuri effetti sconosciuti dei vaccini	○	○	○	○	○	○

* 39. Riserve riguardo il profitto commerciale

	Fortemente in disaccordo					Fortemente d'accordo
I vaccini producono molto fatturato per le aziende farmaceutiche ma pochi benefici alle persone	○	○	○	○	○	○
Le Autorità promuovono la vaccinazione per profitto, non per la salute delle persone	○	○	○	○	○	○
I programmi di vaccinazione sono un grande complotto	○	○	○	○	○	○

* 40. Preferenza per l’immunità naturale (acquisita successivamente a contrazione di malattia)

	Fortemente in disaccordo					Fortemente d'accordo
L’immunità naturale dura più a lungo di una vaccinazione	◯	◯	◯	◯	◯	◯
L’esposizione naturale a virus e batteri offre la protezione più sicura	◯	◯	◯	◯	◯	◯
Essere naturalmente esposti alle malattie è più sicuro per il sistema immunitario che essere esposti attraverso la vaccinazione	◯	◯	◯	◯	◯	◯

Sezione alfabetizzazione sanitaria - Compilazione da parte dei **Genitori**

* 41. L'elenco sottostante contiene parole mediche e parole che possono sembrare tali, ma non lo sono. Barri solo le caselle relative a parole che secondo Lei SONO termini medici. NON consulti Internet o altre fonti e NON si faccia aiutare da altre persone!

- ☐ Antitetranico
- ☐ Epatite
- ☐ Polmonite
- ☐ Respingente
- ☐ Zoster
- ☐ Dittero
- ☐ Posologia
- ☐ Asma
- ☐ Polmomielite
- ☐ Infiammatorio
- ☐ Sifilide
- ☐ Anemia
- ☐ Controindicazioni
- ☐ Allegrene
- ☐ Calendario
- ☐ Gravidismo
- ☐ Antibiogramma
- ☐ Stress
- ☐ Allegoria
- ☐ Ellargico
- ☐ Parenterale
- ☐ Poziente
- ☐ Chemioterapia
- ☐ Gravidanza
- ☐ Occitanica
- ☐ Diagnosi
- ☐ Nausia
- ☐ Limpociti
- ☐ Impetigine
- ☐ Ittero
- ☐ Prescrizione
- ☐ Linfonodi
- ☐ Aborigeno
- ☐ Asplenia
- ☐ Convulsioni
- ☐ Avvertenze
- ☐ Resistenza
- ☐ Amoxacellina
- ☐ Pelvice
- ☐ Rottovirus

- [ ] Parentale
- [ ] Disinfezione
- [ ] Pandomico
- [ ] Equipollente
- [ ] Testino
- [ ] Maloria
- [ ] Meningococco
- [ ] Papillomavirus
- [ ] Pustola
- [ ] Alcolioso
- [ ] Cerpes
- [ ] Antibiotici
- [ ] Batterio
- [ ] Antiregressivo
- [ ] Anafilassi
- [ ] Vaccillaneo
- [ ] Dose
- [ ] Eruzione
- [ ] Orale
- [ ] Germi
- [ ] Varicella
- [ ] Gonorrea
- [ ] Somministrazione
- [ ] Tumico
- [ ] Locazione
- [ ] Immunizzazione
- [ ] Insonniaco
- [ ] Fungo
- [ ] Autista
- [ ] Contrapazione

Conoscenze, Atteggiamenti e Comportamenti - Compilazione da parte dei **Genitori**

Questa parte del questionario prevede delle domande su conoscenze/atteggiamenti/comportamenti verso le vaccinazioni e le malattie prevenibili.

* 42. Per favore, spunti la risposta più vicina al Suo pensiero.

	Assolutamente sì	Molto	Abbastanza	Poco	Per niente
Sa cosa sono i vaccini?	○	○	○	○	○
È favorevole alla vaccinazione in età pediatrica?	○	○	○	○	○
È favorevole alla vaccinazione in età adulta?	○	○	○	○	○
Sa quali sono i vaccini disponibili oggi per la popolazione italiana?	○	○	○	○	○
Conosce dove e chi somministra i vaccini?	○	○	○	○	○
Secondo Lei i vaccini hanno delle controindicazioni?	○	○	○	○	○
Si possono contrarre malattie anche se si è stati vaccinati?	○	○	○	○	○

	Assolutamente sì	Molto	Abbastanza	Poco	Per niente
La vaccinazione è efficace anche dopo aver contratto l'infezione o essere stati a contatto con un caso contagioso?	○	○	○	○	○
Ha mai sentito parlare di Papilloma Virus Umano (HPV)?	○	○	○	○	○
Pensa che l'HPV sia pericoloso?	○	○	○	○	○
Conosce le conseguenze dell'infezione HPV-correlata?	○	○	○	○	○
Ha mai sentito parlare di vaccinazione e prevenzione contro l'HPV?	○	○	○	○	○
Pensa che sia alta la possibilità di contrarre l'infezione?	○	○	○	○	○

Conoscenze sul Virus HPV - Compilazione da parte dei **Genitori**

Questo questionario mira a valutare le conoscenze sul virus dell'HPV e delle malattie a esso correlate.

* 43. Per ogni affermazione sotto si prega di scegliere tra “Vero”, “Falso” e “Non so”. Se non hai mai sentito parlare dell’HPV, selezioni “Non so"

	Vero	Falso	Non so
Solo le donne possono contrarre l’HPV	○	○	○
L’HPV può provocare tumore cervicale nelle donne	○	○	○
L’HPV può provocare tumore in aree come la testa e il collo	○	○	○
L’HPV causa il tumore solo nelle donne	○	○	○
L’HPV può causare verruche genitali	○	○	○
Una persona può avere l’HPV per anni senza accorgersene	○	○	○

	Vero	Falso	Non so
L'HPV viene trasmesso tramite il sesso	○	○	○
Molte persone positive all'HPV hanno segni o sintomi visibili della malattia	○	○	○
La probabilità di contrarre l'HPV per una persona aumenta con l'aumentare dei suoi partner sessuali	○	○	○
Quasi tutte le persone sessualmente attive contrarranno l'HPV a un certo punto	○	○	○
Il vaccino per l'HPV è raccomandato solo per le donne	○	○	○
Per raggiungere la protezione totale contro l'HPV è necessaria più di una dose di vaccino	○	○	○
Il vaccino per HPV è più efficace se somministrato a persone che non hanno mai fatto sesso	○	○	○
