# Supplementary material for: Health literacy among early adolescents in Umbria, Italy: a cross-sectional study
Source: Front Public Health. 2026 Oct 27;14:1925432. doi: 10.3389/fpubh.2026.1925432 (PMC13748303; doi:10.3389/fpubh.2026.1925432)
Supplement: Supplementary file 2 [file Data_Sheet_2.PDF]

Table S2. Item-level descriptive and discrimination statistics for the Italian MOHLAA-Q

Scale	Item	Item mean	Item SD	Corrected item-total r	Item-total flag	Difficulty (Scale D)
A	ease_of_accessing_information_num	2.953	0.552	0.359	Acceptable	
A	understanding_information_leaflets_num	2.488	0.751	0.382	Acceptable	
A	evaluating_media_information	2.481	0.723	0.422	Acceptable	
A	following_doctors_instructions	3.244	0.529	0.354	Acceptable	
A	information_on_psychological_problems	2.687	0.723	0.432	Acceptable	
A	protection_against_sexually_transmitted_diseases	2.727	0.734	0.419	Acceptable	
A	trust_in_media	2.559	0.647	0.540	Acceptable	
A	physical_activity_recommendations	3.277	0.555	0.398	Acceptable	
A	information_on_healthy_behaviors	3.218	0.554	0.502	Acceptable	
A	information_on_food_products	2.926	0.689	0.464	Acceptable	
A	information_on_health_effects	2.968	0.576	0.513	Acceptable	
A	promotion_of_a_healthy_environment	2.924	0.656	0.392	Acceptable	
B	last_doctor_visit	3.106	0.791	0.350	Acceptable	
B	avoided_behaviors	3.156	0.850	0.465	Acceptable	
B	help_from_siblings_and_friends	3.062	0.748	0.409	Acceptable	
B	talking_about_health	3.535	0.644	0.277	Weak (<0.30)	
C	attention_to_health	3.312	0.626	0.431	Acceptable	
C	advice_about_illness	4.062	0.986	0.095	Weak (<0.30)	
C	protection_against_illness	4.104	0.821	0.293	Weak (<0.30)	
C	feeling_at_ease	4.185	0.801	0.332	Acceptable	
C	importance_of_being_informed	4.185	0.793	0.445	Acceptable	
D	score_for_question_1_scale_D	0.076	0.266	-0.038	Negative: check scoring	0.076
D	score_for_question_2_scale_D	0.641	0.480	0.109	Weak (<0.30)	0.641
D	score_for_question_3_scale_D	0.388	0.488	0.101	Weak (<0.30)	0.388
D	score_for_question_4_scale_D	0.509	0.501	0.126	Weak (<0.30)	0.509
D	score_for_question_5_scale_D	0.306	0.461	0.118	Weak (<0.30)	0.306
D	score_for_question_6_scale_D	0.656	0.476	0.036	Weak (<0.30)	0.656
D	score_for_question_7_scale_D	0.274	0.446	0.029	Weak (<0.30)	0.274
D	score_for_question_8_scale_D	0.641	0.480	0.069	Weak (<0.30)	0.641

Note. Corrected item-total correlations correlate each item with the sum of the remaining items in its scale. For dichotomous Scale D items, this is the corrected point-biserial correlation; difficulty is the proportion correct.

Table S3. Fit indices and standardized factor loadings for the correlated four-factor model

Factor	Item	Standardized loading	p-value	Flag
Factor_A	ease_of_accessing_information_num	0.476	<0.001	OK
Factor_A	understanding_information_leaflets_num	0.502	<0.001	OK
Factor_A	evaluating_media_information	0.523	<0.001	OK
Factor_A	following_doctors_instructions	0.498	<0.001	OK
Factor_A	information_on_psychological_problems	0.543	<0.001	OK
Factor_A	protection_against_sexually_transmitted_diseases	0.556	<0.001	OK
Factor_A	trust_in_media	0.686	<0.001	OK
Factor_A	physical_activity_recommendations	0.554	<0.001	OK
Factor_A	information_on_healthy_behaviors	0.696	<0.001	OK
Factor_A	information_on_food_products	0.556	<0.001	OK
Factor_A	information_on_health_effects	0.741	<0.001	OK
Factor_A	promotion_of_a_healthy_environment	0.516	<0.001	OK
Factor_B	last_doctor_visit	0.563	<0.001	OK
Factor_B	avoided_behaviors	0.652	<0.001	OK
Factor_B	help_from_siblings_and_friends	0.683	<0.001	OK
Factor_B	talking_about_health	0.432	<0.001	OK
Factor_C	attention_to_health	0.718	<0.001	OK
Factor_C	advice_about_illness	0.213	0.001	Weak loading (<0.30)
Factor_C	protection_against_illness	0.436	<0.001	OK
Factor_C	feeling_at_ease	0.578	<0.001	OK
Factor_C	importance_of_being_informed	0.682	<0.001	OK
Factor_D	score_for_question_1_scale_D	0.101	0.346	Weak loading (<0.30)
Factor_D	score_for_question_2_scale_D	0.109	0.176	Weak loading (<0.30)
Factor_D	score_for_question_3_scale_D	-0.109	0.212	Weak loading (<0.30)
Factor_D	score_for_question_4_scale_D	0.124	0.129	Weak loading (<0.30)
Factor_D	score_for_question_5_scale_D	0.080	0.314	Weak loading (<0.30)
Factor_D	score_for_question_6_scale_D	-0.077	0.351	Weak loading (<0.30)
Factor_D	score_for_question_7_scale_D	0.345	0.004	OK
Factor_D	score_for_question_8_scale_D	0.291	0.008	Weak loading (<0.30)

Table S4. Assessment of the proportional-odds assumption for the ordinal logistic regression models

Scale	Predictor	Chi-square	p-value	Assessment
**A**	Sex	6.72	0.035	**Violated**
**A**	Province	1.46	0.481	Not violated
**A**	Parentage	0.40	0.819	Not violated
**A**	Responding parent's educational attainment	2.37	0.307	Not violated
**A**	Spouse/cohabitant's educational attainment	3.16	0.206	Not violated
**A**	Responding parent's employment status	-	-	Indeterminate due to separation
**A**	Spouse/cohabitant's employment status	2.65	0.266	Not violated
**A**	Responding parent's citizenship	-	-	Model not estimated
**A**	Responding parent's religious orientation	3.09	0.543	Not violated
**A**	Parental health literacy - IMETER	3.83	0.430	Not violated
**A**	Responding parent's political orientation	-	-	Indeterminate due to separation
**B**	Sex	8.06	0.018	**Violated**
**B**	Province	0.22	0.895	Not violated
**B**	Parentage	0.46	0.795	Not violated
**B**	Responding parent's educational attainment	0.21	0.901	Not violated
**B**	Spouse/cohabitant's educational attainment	0.46	0.796	Not violated
**B**	Responding parent's employment status	0.05	0.975	Not violated
**B**	Spouse/cohabitant's employment status	0.61	0.738	Not violated
**B**	Responding parent's citizenship	-	-	Model not estimated
**B**	Responding parent's religious orientation	7.79	0.100	Not violated
**B**	Parental health literacy - IMETER	4.02	0.403	Not violated
**B**	Responding parent's political orientation	7.65	0.105	Not violated
**C**	Sex	0.13	0.720	Not violated
**C**	Province	0.02	0.895	Not violated
**C**	Parentage	0.07	0.792	Not violated
**C**	Responding parent's educational attainment	0.24	0.627	Not violated
**C**	Spouse/cohabitant's educational attainment	0.15	0.697	Not violated
**C**	Responding parent's employment status	-	-	Indeterminate due to separation
**C**	Spouse/cohabitant's employment status	0.41	0.521	Not violated
**C**	Responding parent's citizenship	-	-	Model not estimated
**C**	Responding parent's religious orientation	1.80	0.406	Not violated
**C**	Parental health literacy - IMETER	0.76	0.684	Not violated
**C**	Responding parent's political orientation	-	-	Indeterminate due to separation
**D**	Sex	0.33	0.567	Not violated
**D**	Province	0.36	0.548	Not violated
**D**	Parentage	0.02	0.895	Not violated
**D**	Responding parent's educational attainment	0.19	0.663	Not violated
**D**	Spouse/cohabitant's educational attainment	3.57	0.059	Not violated
**D**	Responding parent's employment status	0.09	0.761	Not violated
**D**	Spouse/cohabitant's employment status	0.02	0.892	Not violated
**D**	Responding parent's citizenship	-	-	Model not estimated
**D**	Responding parent's religious orientation	1.82	0.403	Not violated
**D**	Parental health literacy - IMETER	0.62	0.732	Not violated
**D**	Responding parent's political orientation	-	-	Indeterminate due to separation

Note. The proportional-odds assumption was evaluated using a cluster-robust Brant-type Wald test based on cumulative binary logistic regressions, retaining the same complete-case samples, eligible predictor categories, and school-level clustering used in the primary analyses. A p-value <0.05 was considered evidence against the assumption. 'Indeterminate due to separation' indicates that at least one diagnostic binary model was affected by complete or quasi-complete separation and produced an unreliable estimate. 'Model not estimated' indicates that fewer than two eligible predictor categories remained after categories with fewer than 10 complete observations were excluded. Statistics and p-values from unreliable diagnostic models are not reported.
